# Global, regional, and national burden of cervical cancer for 195 countries and territories, 2007–2017: findings from the Global Burden of Disease Study 2017

**DOI:** 10.1186/s12905-021-01571-3

**Published:** 2021-12-18

**Authors:** Miaomiao Zhao, Qunhong Wu, Yanhua Hao, Jingcen Hu, Yuexia Gao, Shan Zhou, Liyuan Han

**Affiliations:** 1 Department of Health Management, School of Public Health, Nantong University, Nantong, Jiangsu China; 2 Department of Social Medicine, School of Health Management, Harbin Medical University, Harbin, Heilongjiang China; 3 Department of Global Health, Hwa Mei Hospital, University of Chinese Academy of Sciences, Ningbo, Zhejiang China; 4 Department of Global Health, Ningbo Institute of Life and Health Industry, University of Chinese Academy of Sciences, Ningbo, 315010 Zhejiang China; 5 Department of Endocrinology, Hwa Mei Hospital, University of Chinese Academy of Sciences, Ningbo, Zhejiang China

**Keywords:** Global Burden of Disease, Cervical cancer, Human papillomavirus, Incidence, Disability-adjusted life-years, Death

## Abstract

**Background:**

Cervical cancer is one of the most common cancers among women worldwide. The formulation or evaluation on prevention strategies all require an accurate understanding of the burden for cervical cancer burden. We aimed to report the up-to-date estimates of cervical cancer burden at global, regional, and national levels.

**Methods:**

Data were extracted from the Global Burden of Diseases, Injuries, and Risk Factors Study (GBD) 2017 study. The counts, age-standardized rates, and percentage changes of incidence, disability-adjusted life-years (DALYs), and death attributed to cervical cancer at the global, regional, and national levels in all 195 countries and territories from 21 regions during 2007 to 2017 by age and by Socio-demographic Index (SDI) were measured. All estimates were reported with 95% uncertainty intervals (UIs).

**Results:**

In 2017, 601,186 (95% UI 554,455 to 625,402) incident cases of cervical cancer were reported worldwide, which caused 8,061,667 (7,527,014 to 8,401,647) DALYs and 259,671 (241,128 to 269,214) deaths. The age-standardized rates for incidence, DALYs and death decreased by − 2.8% (− 7.8% to 0.6%), − 7.1% [− 11.8% to − 3.9%] and − 6.9% [− 11.5% to − 3.7%] from 2007 to 2017, respectively. The highest age-standardized incidence, DALYs and death rates in 2017 were observed in the low SDI quintile, Oceania, Central and Eastern Sub-Saharan Africa. During 2007 to 2017, only East Asia showed increase in these rates despite not significant. At the national level, the highest age-standardized rates for incidence, DALYs, and death in 2017 were observed in Kiribati, Somalia, Eritrea, and Central African Republic; and Georgia showed the largest increases in all these rates during 2007 to 2017.

**Conclusion:**

Although the age-standardized rates for incidence, DALYs, and death of cervical cancer have decreased in most parts of the world from 2007 to 2017, cervical cancer remains a major public health concern in view of the absolute number of cervical cancer cases, DALYs, and deaths increased during this period. The challenge is more prone to in the low SDI quintile, Oceania, Central and Eastern Sub-Saharan Africa, East Asia, and some countries, suggesting an urgent to promote human papillomavirus vaccination in these regions.

**Supplementary Information:**

The online version contains supplementary material available at 10.1186/s12905-021-01571-3.

## Background

Cervical cancer is the fourth most frequently diagnosed cancer, and the fourth leading cause of cancer deaths in women. According to the Global Cancer Incidence, Mortality and Prevalence (GLOBOCAN), approximately 570,000 cases and 311,000 deaths of cervical cancer were estimated to have occurred in 2018 worldwide [1]. Without significant intervention, the global burden of cervical cancer is expected to increase to nearly 700,000 cases and 400,000 deaths by 2030, representing a 21% and 27% increase in the number of cases and deaths, respectively [2].

Persistent infection with high-risk types of human papillomavirus (hrHPV) is a necessary cause of cervical cancer [3, 4]. Compelling evidence confirmed that HPV vaccination programs for the most common hrHPV would prevent approximately 87% of cervical cancer cases worldwide [5]. Since HPV vaccination was licensed in 2006, approximately 80 countries and territories implemented national HPV vaccination programs, covering more than 100 million women [6]. In 2018, the World Health Organization issued a global call to eliminate cervical cancer as a public health problem by this century [7]. Now more than ever, effective cervical cancer control planning requires an accurate estimation of this disease.

Although studies have reported the estimates of cervical cancer burden, they are limited to confined regions or countries or used literature review methods [8, 9]. The GLOBOCAN project regularly provides the estimates of the global incidence and mortality of cervical cancer; however, it does not provide estimates the temporal and geographical trends or for disability-adjusted life-years (DALYs) [1, 10]. DALYs is a useful composite metric that accounts for both the mortality and morbidity associated with a disease and enables contextualization of the disease burden through cross-disease and cross-geographic comparisons [11]. Moreover, no study has investigated the association between cervical cancer burden estimates and SDI at regional level.

The Global Burden of Diseases, Injuries, and Risk Factors Study (GBD) uses a unique approach to generate estimates for all 195 countries and territories using a wide range of data sources [11–13]. In this study, we used data from the GBD 2017 to estimate the global, regional, and national-specific counts, age-standardized rates, and percentage changes for incidence, DALYs, and death of cervical cancer, and their temporal and geographical trends in all 195 countries and territories by age and the SDI for the period 2007–2017. In addition, we used the SDI to identify areas with cervical cancer burden better or worse than expected. The comparison of the cervical cancer metrics among different countries or regions would provide policymakers with the required information regarding their secular trends and gaps with expected levels to allocate resources appropriately.

## Method

### Overview

The data we used were from the GBD 2017, which reports the comparative assessment of health loss and associated risk factors for 282 causes of death, 354 causes of years lived with disability (YLDs), and 359 causes of DALYs in 195 countries and territories from 1990 to 2017. Detailed descriptions of the method and approach of the GBD 2017 have been published elsewhere [11–13]. The locations included in the GBD 2017 were arranged into a set seven super-regions and a further nested set of 21 regions [14]. The GBD study was performed in accordance with the Guidelines for Accurate and Transparent Health Estimates Reporting.

### Data sources

Data related to cervical cancer disease burden were obtained from the Global Health Data Exchange database. The GBD 2017 study used all available health data sources through comprehensive searches and summarizes of published and unpublished data.

### Estimation of cervical cancer burden

In the GBD 2017, mortality estimate was first estimated in the process of cancer burden estimation. The cancer incidence data from cancer registry was transformed to mortality estimates through the model mortality-to-incidence ratios (MIRs). The cancer incidence estimate was computed by dividing the final mortality estimates by the MIR for each cancer type, sex, age group, location, and year to estimate cancer incidence. Detailed descriptions of the cancer mortality and incidence estimates using in the GBD 2017 have been published elsewhere [15–17].

In the GBD, comparisons between diseases were performed based on DALYs. DALYs are the sum of YLDs and years of life lost (YLLs), thereby incorporating both fatal and nonfatal burden [11]. YLDs are calculated as multiplying the prevalence of a disease or sequela by their corresponding disability weight range from 0 (“no health loss”) to 1 (“dead”) [12]. YLLs, the measure of premature death, are calculated as the sum of each death counts multiplied by the standard life expectancy at each age [13].

Here, we report the estimates of cervical cancer incidence, DALYs, and deaths from 2007 to 2017. All rates are presented as per 100,000 person-years and all estimates are reported with 95% uncertainty intervals (UIs).

### SDI

The SDI is a compound measure of social-demographic development status for each location, calculated as the mean of the scaled values of the total fertility rate in women under the age of 25 years, educational attainment in women over aged 15 years, and lag-distributed income per capita [12]. The SDI value ranges from 0 (worst) to 1 (best). Five quintiles were used to categorize and present in the GBD 2017 results: low, low-middle, middle, high-middle, and high quintile. In addition, we analyzed the association of the SDI value with the age-standardized rates for incidence, DALYs, and death of cervical cancer for the GBD regions from 1990 to 2017.

All figures in this study were drawn using R software (version 3.6.3).

## Results

### Incidence counts, age-standardized incidence rates per 100,000 population, and percentage changes by countries and territories

**Fig. 1:**
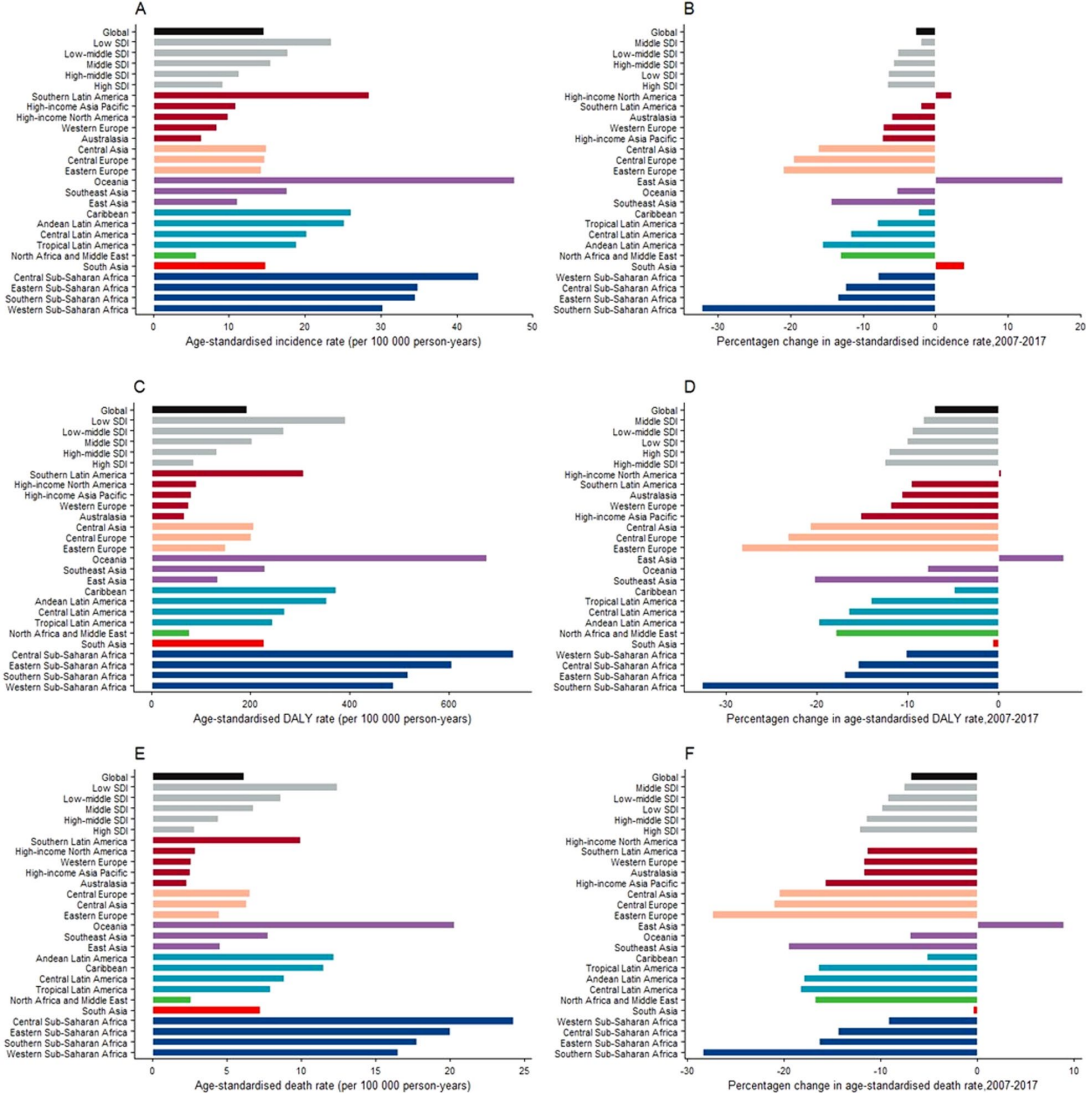
Age-standardized rates for incidence (**A**), DALYs (**C**), and death (**E**) of cervical cancer in 2017, and percentage change of age-standardized rates for incidence (**B**), DALYs (**D**), and death (**F**) of cervical cancer between 2007 and 2017, globally, by SDI quintile and by 21 GDB regions. *DALYs* disability-adjusted life-years, *SDI* socio-demographic index

In 2017, approximately 601,186 incident cases of cervical cancer were reported globally (95% UI, 554,455 to 625,402), with an 18.9% (12.8% to 23.0%) increase since 2007 (Additional file 1: Table S1). The age-standardized incidence rate of cervical cancer was 14.5 (13.4 to 15.1) per 100,100 person-years in 2017, with a − 2.8% (− 7.8% to 0.6%) decrease since 2007 (Fig. 1A, B).

In 2017, the highest age-standardized incidence rates of cervical cancer were observed in the low SDI quintile (23.5 [21.3 to 26.0] per 100,000 person-years), whereas the lowest rate was observed in the high SDI quintile (9.2 [8.8 to 9.5]) (Fig. 1A). The age-standardized incidence rates decreased in all the SDI quintiles from 2007 to 2017, with the highest decrease in the high SDI quintile (− 6.7% [− 10.1% to − 3.5%]) and the lowest decrease in the middle SDI quintile (− 2.0% [− 11.0% to 3.3%]) (Fig. 1B).

Regionally, the highest age-standardized incidence rates of cervical cancer were found in Oceania (47.6 [34.8 to 62.8] per 100,000 person-years), Central Sub-Saharan Africa (42.9 [31.5 to 52.8]), and Eastern Sub-Saharan Africa (34.9 [29.8 to 41.4]), while the lowest incidence rates were found in North Africa and Middle East (5.6 [4.9 to 6.2] per 100,000 person-years), Australasia (6.3 [5.3 to 7.4]), and Western Europe (8.3 [7.8 to 8.8]) (Fig. 1A). During 2007 to 2017, an increase in incidence rates was noted only in East Asia (17.6% [− 10.9% to 27.8%]), South Asia (4% [− 4.3% to 12.5%]) and high-income North America (2.2% [− 3.8% to 8.7%]), but the increase was not significant. Southern Sub-Saharan Africa had the largest decrease in age-standardized incidence rate (− 32.3% [− 41.6% to − 24.0%]), followed by Eastern Europe (− 21.1% [− 26.0 to − 15.5%]) and Central Europe (− 19.6% [− 24.1% to − 14.8%]) (Fig. 1B).

**Fig. 2:**
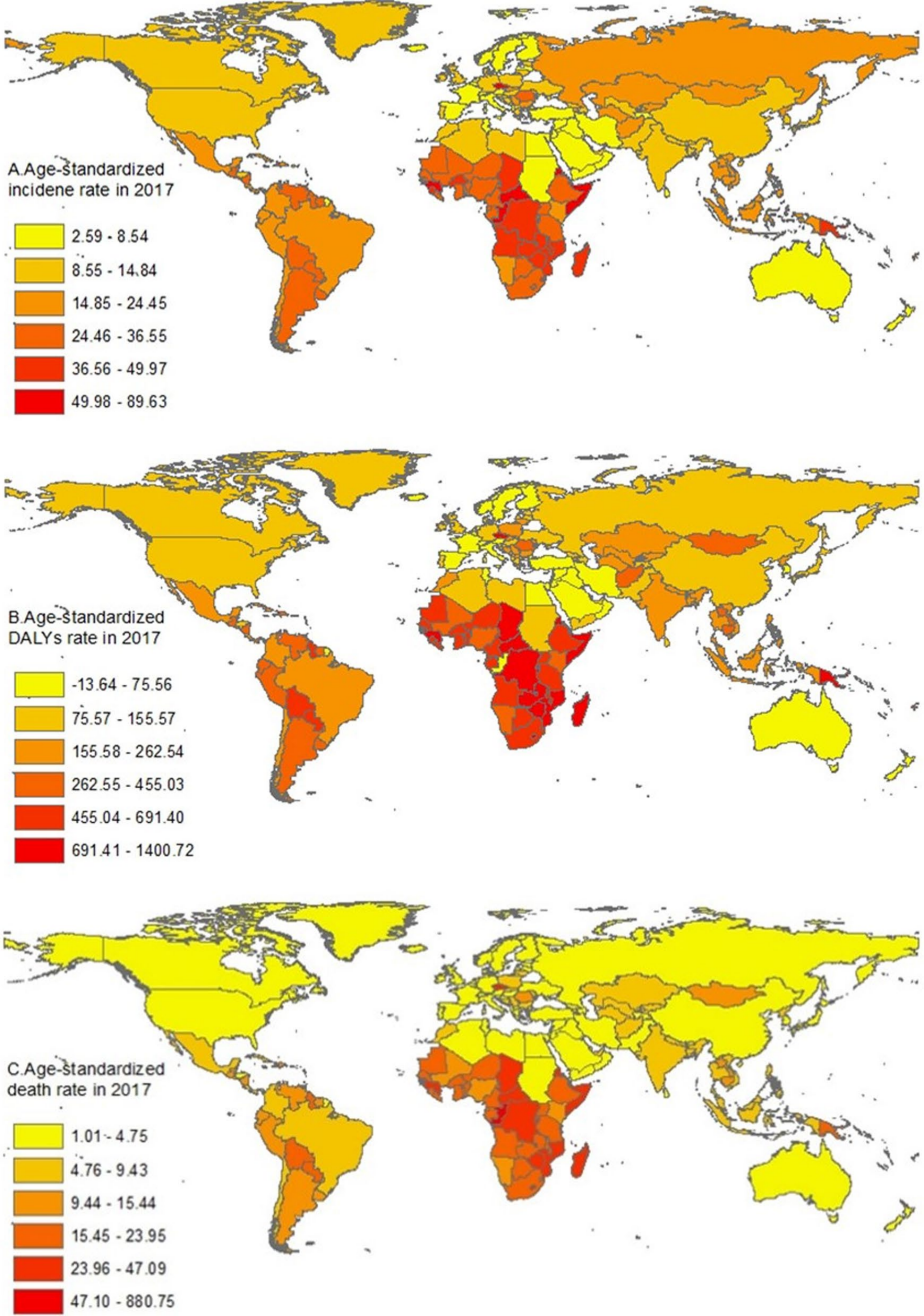
Age-standardize rates for incidence (**A**), DALYs (**B**) and death (**C**) of cervical cancer across 195 countries and territories, 2017. This figure was drawn using the extracted data by the authors. *DALYs* disability-adjusted life-years

The 2017 global map of the age-standardized incidence rates of cervical cancer is presented in Fig. 2A. In 2017, the highest age-standardized incidence rates were found in Kiribati (89.6 [65.5 to 118.6] per 100,000 person-years), Somalia (56.6 [37.4 to 82.1]), and Eritrea (56.2 [39.4 to 78.5]). On the contrary, the lowest incidence rates were found in Kuwait (2.6 [2.2 to 3.1] per 100,100 person-years), Iraq (2.7 [2.3 to 3.3]), and Egypt (3.2 [2.5 to 4.0]). During 2007 to 2017, the largest increases in age-standardized incidence rates were noted in Georgia (27.9% [10.6% to 48.3%], China (19.7% [− 10.3% to 30.5%]) and Costa Rica (15.2% [− 2.3% to 35.7%]), and the largest decreases were noted in Kuwait (− 44.7% [− 53.8% to − 33.8%]), Lithuania (− 42.0% [− 50.2% to − 31.6%]), and Jordan (− 36.2% [− 50.8% to − 14.2%])(Additional file 2: Table S2).

The 2017 age-specific (15 to > 80 years) incidence rates of cervical cancer worldwide and by all the SDI quintile are presented in Additional File 3: Fig. S1A. Globally, the incidence rates of cervical cancer increased after the age of 25 years, peaked at the age of 50–54 years, and decreased slightly thereafter. Generally, the peak incidence rate was earlier in the lower SDI quintiles such as at the age of 50–54 years in the low SDI quintile and at 65–69 years in the high-middle SDI quintile. In the high SDI quintile, the incidence rate increased until the age of 35–39 years and was stable thereafter until a further rise after the age of 75 years and more.

**Fig. 3:**
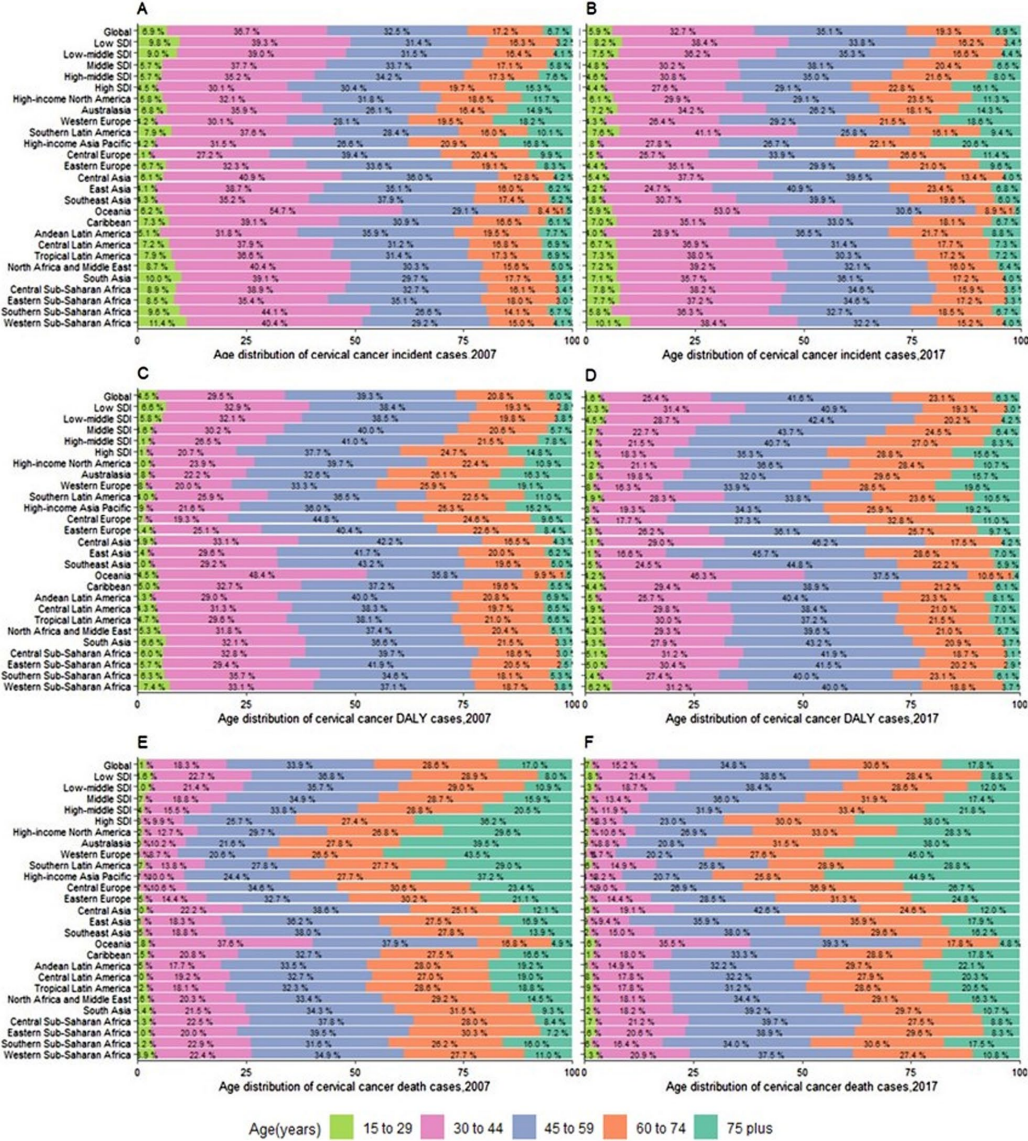
Age-distribution for incident, DALYs, and death cases of cervical cancer by 21GBD regions, 2007 and 2017. **A** Incident cases of cervical cancer in 2007. **B** Incident cases of cervical cancer in 2017. **C** DALYs cases s of cervical cancer in 2007. **D** DALYs cases of cervical cancer in 2017. **E** Death cases of cervical cancer in 2007. **F** Death cases of cervical cancer in 2017. *DALYs* disability-adjusted life-years, *SDI* socio-demographic index

Figure 3A, B illustrate the age distribution of cervical cancer incident cases in 2007 and 2017. In 2017, the lower the SDI, the higher the proportion of incident cases among younger women (≤ 44 years), with the highest proportion of incident cases among younger women in Oceania (58.9%), Southern Latin America (48.7%), and Western Sub-Saharan Africa (48.5%). During 2007 to 2017, a slight decrease was noted in the proportion of incident cases among younger women worldwide, in all the SDI quintiles and in most regions, except for South and Tropical Latin America, Eastern Europe, and Eastern Sub-Sahara Africa.

**Fig. 4:**
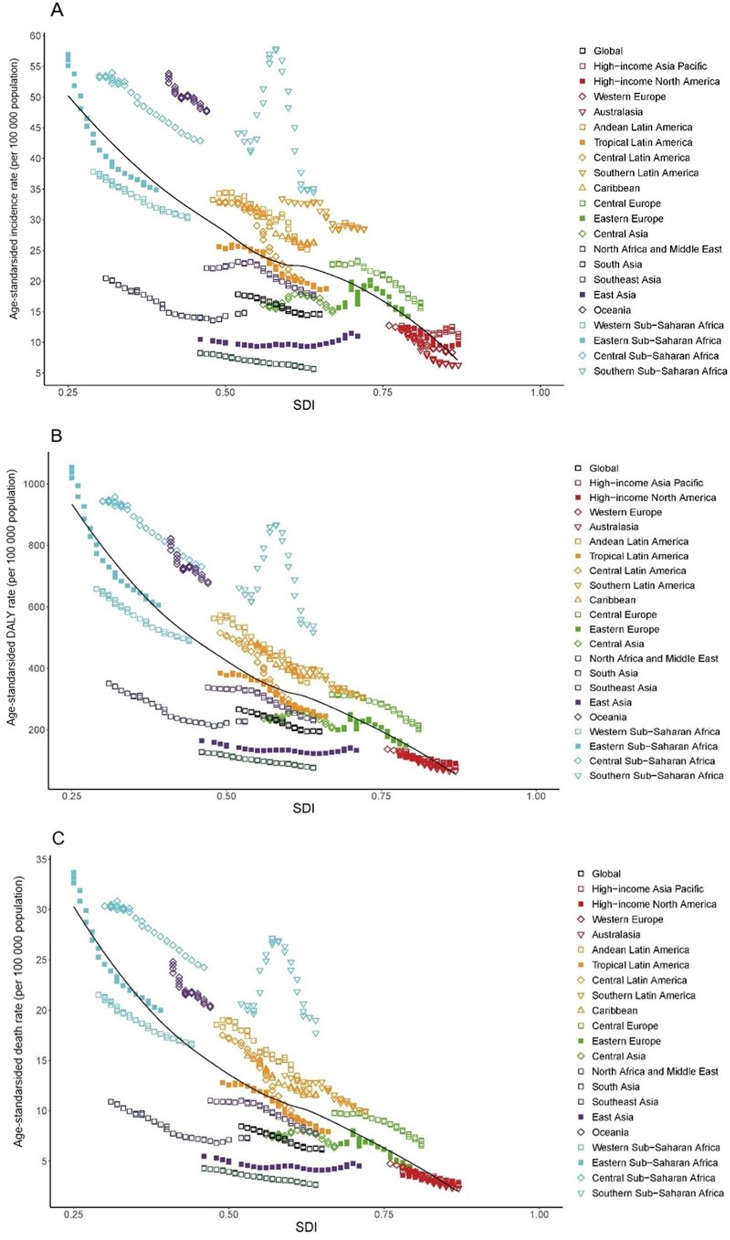
The trend in age-standardized incidence (**A**), DALYs (**B**), and death (**C**) rates of cervical cancer in 21 GBD regions by SDI, 1990–2017. For each region, points from left to right depict estimates from each year from 1990 to 2017. The black line represents the average expected relationship between SDI and burden estimates rates for T2DM based on values from each geographical region over the 1990–2017 estimation period. *DALYs* disability-adjusted life-years, *SDI* socio-demographic index

Figure 4A illustrates the secular trend in age-standardized incidence rate of cervical cancer across the SDI quintiles by regions from 1990 to 2017 and the expected levels based only on the SDI values of the global regions. We found that the expected values of the age-standardized incidence rate decreased with an increase in the SDI value. Most regions generally saw a steady decrease in incidence rate with an increase in the SDI value, with values close to the expected line. In addition, the highest observed value was corresponded to an SDI of 0.58 in Southern Sub-Saharan Africa, and, observed values largely decreased with increasing SDI value after 2007 in this region. East Asia exhibited a slight increase in the observed age-standardized incidence rate with increasing SDI value. Eastern sub-Saharan Africa exhibited the largest decrease in incidence rate with an increase in the SDI value.

### The DALYs counts and age-standardized DALY rates per 100,000 population, and their percentage changes by countries and territories

In 2017, cervical cancer was responsible for 8,061,667 [7,527,014 to 8,401,647] DALYs globally, representing a 15.2% [9.5% to 19.2%] increase in DALYs since 2007 (Additional file 1: Table S1). The 2017 age-standardized DALYs rate was 193.0 [180.2 to 201.2] per 100,000 person-years, which had declined by − 7.1% [− 11.8% to − 3.9%] from 2007 to 2017 (Fig. 1C, D).

In 2017, the low SDI quintile had the highest age-standardized DALYs rate (391.2 [352.7 to 431.5] per 100,000 person-years), whereas the high SDI quintile had the lowest DALYs rate (84.7 [81.9 to 87.8]) (Fig. 1C). The age-standardized DALYs rates decreased in all the SDI quintiles from 2007 to 2017, with the largest decrease in the high-middle SDI quintile (− 12.5% [− 22.7% to − 8.2%]) and the lowest decrease in the middle SDI quintile (− 8.3% [− 16.5% to − 3.8%]) (Fig. 1D).

A substantial difference was noted in the age-standardized DALYs rates across regions, ranging from the highest rates in Central Sub-Saharan Africa (730.8 [544.9 to 900.8] per 100,000 person-years), Oceania (676.0 [507.9 to 867.4]), and Eastern Sub-Saharan Africa (605.8 [514.8 to 722.7]) to the lowest rates in Australasia (65.4 [56.8 to 75.1] per 100,000 person-years), Western Europe (74.7 [70.6 to 79.0]), and North Africa and Middle East (75.7 [65.1 to 83.9]) (Fig. 1C). During 2007 to 2017, an increase in age-standardized DALYS rates were observed only in high-income East Asia (7.2% [− 19.7% to 15.8%]) and high-income North America (0.2% [− 4.7% to 5.6%]). In this period, the largest decreases in the age-standardized DALYs rates were noted in Southern Sub-Saharan Africa (− 32.7% [− 40.4% to − 25.9%]), Eastern Europe (− 28.3% [− 31.0% to − 25.5%]), and Central Europe (− 23.3% [− 27.0% to − 19.3%]) (Fig. 1D).

The global map of age-standardized DALYs rates of cervical cancer in 2017 is presented in Fig. 2B. The 2017 age-standardized DALYs rates varied by nearly 50 times across countries, with the highest DALYs rates in Kiribati (1400.7 [1040.1 to 1828.6] per 100,000 person-years), Central African Republic (1064.6 [638.6 to 1487.8]), and Somalia (1062.1 [716.1 to 1546.9]), while the lowest DALYs rates in Kuwait (29.1 [25.4 to 33.2] per 100,000 person-years), Iraq (39.0 [32.5 to 46.6]), and Egypt (42.4 [33.8 to 52.1]) (Fig. 2B). From 2007 to 2017, the largest increases in the age-standardized DALYs rates were noted in Georgia (25.5% [11.8% to 41.1%]), Tajikistan (10.5% [− 11.2% to 34.6%]), Jamaica (10.2% [− 13.3% to 41.0%]). On the contrary, the largest decreases were noted in Kuwait (− 48.7% [− 55.5% to − 40.7%]), Lithuania (− 44.1% [− 36.2% to − 50.6%]), and Jordan (− 40.5% [− 20.8% to − 53.9%]) (Additional file 2: Table S2).

The 2017 age-specific (15 to > 80 years) DALYs rates of cervical cancer worldwide and by the SDI quintile are presented in Additional file 3: Fig. S1B. The DALYs rates peaked at the age of 50–54 years globally as well as in the most SDI quintiles and at 55–59 years in the high SDI quintile.

Figure 3C, D illustrate the age distribution of DALYs for cervical cancer in 2007 and 2017. In 2017, the proportion of DALYs increased in younger women (≤ 44 years) with a decrease in the SDI value, with the highest proportion of DALYs among younger women in Oceania (50.5%), Western Sub-Saharan Africa (47.4%), and Eastern Sub-Saharan Africa (36.3%). During 2007 to 2017, a slight decrease was noted in the proportion of DALYs among younger women worldwide, in all the SDI quintiles and in most regions, except for South Latin America and Eastern Sub-Sahara Africa.

Figure 4B demonstrates the secular trend in the age-standardized DALYs rate across the SDI quintiles by region from 1990 to 2017 and the expected levels based only on the SDI values of the global regions. The correlation of the SDI with age-standardized DALYs rate yielded a pattern similarly to that with the age-standardized incidence rate. What is different is that, the highest observed value was corresponded to an SDI of 0.23 in Eastern Sub-Saharan Africa, while the decrease trend in observed values slowed down with increasing SDI value after 2007 in this region.

### The death counts and age-standardized death rate per 100,000 population, and their percentage changes by country and territory

In 2017, cervical cancer caused 259,671 [241,128 to 269,214] deaths globally, with an age-standardized death rate of 6.2 [5.7 to 6.4] per 100,000 person-years (Additional file 1: Table S1). An 18.8% [12.9% to 22.8%] increase in death cases and a − 6.9% [− 11.5% to − 3.7%] decrease in the age-standardized death rate were observed between 2007 and 2017 (Fig. 1E, F).

In 2017, the low SDI quintile had the highest age-standardized death rate (12.4 [11.3 to 13.8] per 100,000 person-years), whereas the high SDI quintile had the lowest death rate (2.8 [2.8 to 2.9]) (Fig. 1E). Between 2007 and 2017, all the SDI quintiles exhibited a decrease in the age-standardized death rate, with the largest decrease in the high SDI quintile (− 12.2% [− 14.8% to − 9.6%]) and the lowest decrease in the middle SDI quintile (− 7.7% [− 15.7% to − 3.5%]) (Fig. 1F).

In 2017, the highest age-standardized cervical cancer death rates were found in Central Sub-Saharan Africa (24.3 [18.0 to 30.0] per 100,000 person-years), Oceania (20.3 [16.1 to 25.1]), and Eastern Sub-Saharan Africa (20.0 [16.8 to 23.8]), while the lowest death rates were found in Australasia (2.3 [2.0 to 2.7] per 100,000 person-years), Western Europe (2.6 [2.5 to 2.7]), High-income Asia Pacific (2.6 [2.5 to 2.7]), and North Africa and Middle East (2.6 [2.3 to 2.8]) (Fig. 1E). Only East Asia exhibited an increase in the age-standardized death rate during 2007 to 2017 (8.9% [− 17.5% to 17.6%]). Among the other 20 regions with decreased death rates from 2007 to 2017, the largest decreases were noted in Southern Sub-Saharan Africa (− 28.5% [− 35.2% to − 21.0%]), Eastern Europe (− 27.5% [− 30.0% to − 24.8%]), and Central Europe (− 21.1% [− 24.8% to − 17.2%]), and the lowest decreases were noted in high-income North America (− 0.1% [− 4.5% to 4.5%]) and South Asia (− 0.5% [− 7.9% to 7.2%]) (Fig. 1F).

The global map of the age-standardized cervical cancer death rates in 2017 is presented in Fig. 2C. Globally, the 2017 age-standardized death rates varied nearly 50 times across countries. To be specific, the highest age-standardized death rates were found in Kiribati (47.1 [35.3 to 59.6] per 100,000 person-years), Central African Republic (33.9 [20.9 to 45.5]), and Somalia (34.3 [23.4 to 49.9]). On the contrary, the lowest rates were found in Kuwait (1.0 [0.9 to 1.1] per 100,000 person-years), Iraq (1.4 [1.2 to 1.6]), and Saudi Arabia (1.4 [1.2 to 1.8]). From 2007 to 2017, Georgia (27.1% [14.5% to 41.0%]), Guam (11.7% [− 5.9% to 30.7%]) and Tajikistan (11.2% [− 9.3% to 34.5%]) showed the largest increases in the age-standardized death rate. Contrarily, Kuwait (− 48.2% [− 54.6% to − 40.3%]), Lithuania (− 41.5% [− 47.8% to − 33.6%]), and Ukraine (− 38.0% [− 43.4% to − 31.0%]) had the largest decreases in the age-standardized death rate (Additional file 2: Table S2).

The 2017 age-specific (15 to > 80 years) death rates of cervical cancer worldwide and by the SDI quintile are presented in Additional file 3: Fig. S1C. The death rate increased with an in-crease in age globally and in all the SDI quintiles.

Figure 3E, F illustrate the age distribution of cervical cancer death cases in 2007 and 2017. In 2017, the proportion of death cases in younger women (≤ 44 years) in-creased with a decrease in the SDI, with the highest proportion of death cases in younger women in Oceania (38.1%), Western Sub-Saharan Africa (24.2%), and Central Sub-Saharan Africa (23.9%). During 2007 to 2017, a slight decrease was noted in the proportion of death cases in younger women (≤ 44 years) worldwide, in all SDI quintiles and in most regions, except for South Latin America.

Figure 4C demonstrates the secular trend in the age-standardized death rates across the SDI quintiles by regions from 1990 to 2017 and the expected levels based only on the SDI values of the global regions. The correlation of the SDI with the age-standardized death rate yielded a pattern similarly to that with the age-standardized DALYs rate.

## Discussion

This study revealed the most updated temporal and geographical trends of cervical cancer burden at the global, regional, and national level in all 195 countries from 2007 to 2017. Globally, approximately 0.6 million incident cases of cervical cancer were reported in 2017, which caused approximately 8.1 million DALYs and 0.26 million deaths. Between 2007 and 2017, that was 10 years after HPV vaccination was approved, we found although the absolute number of cervical cancer cases, DALYs, and deaths increased, the age-standardized rates for incidence, DALYs, and deaths dropped, during this period.

Our estimates are generally in line with those of the GLOBOCAN estimates. The GLOBOCAN 2018 estimated 0.57 million cervical cancer cases and 0.31 million deaths in 2018 with the age-standardized incidence and death rates of 15.2 and 7.8 per 100,000 person-years [10], presenting an increasing trend in cases and a decreasing trend in the rates [18]. The decreased estimates of the age-standardized incident rate as well as DALYs rate and death rate in our study reflect the increased coverage in HPV vaccination globally, the improvements in genital hygiene, reduced parity, and diminished sexually transmitted disease [19].

We observed that the higher the SDI quintile, the lower the cervical cancer burden. The high SDI quintile were observed the lowest cervical cancer burden, and the largest decrease in cervical cancer burden was mainly attributable to the scaled-up HPV vaccination and population-based cervical cancer screening programs. A systematic review and meta-analysis revealed that a 54%–83% decline in hrHPV infection and a 31%–51% decline in precancerous lesions in young women were observed in high income countries (HICs) with high vaccination coverage [20]. In addition, cervical cytology screening programs with a wide coverage in HICs have achieved 40%–90% reduction in cervical cancer incidence and mortality [21, 22]. However, a challenge faced in the high SDI quintile is the presence of the highest proportion of cervical cancer cases, DALYs, and deaths in older women. Effective vaccination for older women or women with prior HPV infection is necessary in HICs.

The low SDI quintile had the highest cervical cancer burden, showing the highest age-standardized incidence, DALYs, and death rates of cervical cancer, and the highest proportion of cervical cancer cases, DALYs, and deaths in younger women. This is largely related to poverty, lack of resources and infrastructure for cervical cancer screening and treatment [23, 24]. It’s reported that less than 3% women aged 10–20 years in less developed regions and more than 33.6% in more developed regions had received HPV vaccination [6]. Moreover, only approximately 20% of women in less developed regions have ever been screened for cervical cancer compared with more than 60% in more developed regions [25]. Thus, wide coverage of HPV vaccination in low-income regions are an urgent need.

Of the 21 GBD regions analyzed, the highest age-standardized rates for incidence, DALYs, and death of cervical cancer in 2017 were all found in Oceania, Central and Eastern Sub-Saharan Africa. In Oceania, specific data on cervical cancer were not available for some countries [26] and therefore, data were extrapolated from regional estimates and countries with data available [27]. This partially explained the highest burden of cervical cancer in Oceania. In addition, most countries or territories have a low coverage of HPV vaccination and screening programs [28]. Implementation of effective vaccination and screening programs are priorities in these regions.

Sub-Saharan Africa had the highest prevalence of HPV infection worldwide [29, 30]. This partly explain the high burden of cervical cancer in this region. Women in Sub-Saharan Africa have first sexual intercourse and pregnancy at an early age and have high parity, which are the risk factors of HPV infection [31, 32]. Insufficient condom uses and multiple sexual partners are common in Sub-Saharan African women, which are the important enhancers of HPV carcinogenesis [33, 34]. In addition, the high burden of cervical cancer is driven by other factors such as the lack of population-level screening programs, inequitable access to health services, and poverty [29, 35, 36]. On the contrary, North Africa and the Middle East had the lowest cervical cancer burden, and Egypt, Iraq, and Kuwait had the lowest age-standardized rates for incidence and death of cervical cancer. Conservative cultural values for sex might be a critical reason because cervical cancer screening and vaccination programs are lacking in most countries of this region [37, 38]. In addition, the low incidence rate in countries such as Iraq might be attributable to a low detection rate.

Most strikingly, we observed a large increase in the cervical cancer burden in Eastern Asia in 2007 and 2017 despite without significance, which was attributable to China. In 2017, China had the highest numbers of cervical cancer incident cases as well as DAYLs and deaths. Meanwhile, China had the highest increases in age-standardized rates for cervical cancer incidence and death from 2007 to 2017, suggesting a high public health burden of cervical cancer. Then earlier age of sexual debut in women and increased trends of multiple sexual partners and extra-marital relationships in China might contribute to the increased trends [39, 40]. Until now, China approved HPV vaccination for a short time in 2016, with relatively low coverage [41]. The effective control of cervical cancer remains a challenge in China, because HPV vaccination is not covered under medical insurance [42].

At the country level, the highest age-standardized rates for incidence, DALYs, and death of cervical cancer were noted in Kiribati, Somalia, Eritrea, and Central African Republic, while the largest increases in these rates were noted in Georgia, Tajikistan, China, Jamaica, Costa Rica, and Guam, suggesting a consistently high burden of cervical cancer in these countries. Although Pap smears are highly recommended and available for women of childbearing age, screening programs coverage were still low due to lack of organization, limitations of techniques, poor quality control, and insufficient awareness [43–47]. Thus, all the above should be strengthened in these countries.

We found the global cervical cancer incidence rate peaked at the age of 50–54 years, which is consistent with the GLOBACAN study [10]. However, contrary to that study, we found an earlier incidence peak in the least developed regions (low SDI quintile). The age of newly diagnosed cervical cancer patients was younger in the low SDI quintile than in the high SDI quintile, indicating a greater cervical cancer burden in the low SDI quintile. Although age-specific data indicated that cervical cancer could affect women at a wide range of ages, we found that more than 75% of new cases and more than 50% of deaths occurred before the age of 60 years. During 2007 to 2017, the proportion of cervical cancer incident cases, DALYs, and death in elderly women increased, which could be explained by population aging [48].

Potential linear associations between cervical cancer burden and the SDI were observed in most regions. From 1990 to 2017, the age-standardized rates for incidence, DALYs, and death of cervical cancer has decreased with the increasing SDI value. However, these rates were consistently higher than expected in some regions during the past three decades, such as Oceania, Southern and Central Sub-Saharan Africa, Central and Southern Latin America, Caribbean, and Central Europe. Potentially, these regions had worse-than-expected cervical cancer burden, indicating that these regions should be intervention priorities.

According to our knowledge, this study provides an integrated contemporary understanding of the temporal and geographical trends of the global, regional, and national cervical cancer burden by age and SDI in all 195 countries and territories during 2007 to 2017. This study also investigates the association between the SDI and cervical cancer burden, which is helpful in identifying areas where the cervical cancer burden is better or worse than expected. Further, we first used DALYs to describe the cervical cancer burden, which allowed cross-disease and cross-geographic comparisons.

The following limitations should be acknowledged. First, although the GBD 2017 attempted to collect data from all possible sources, data of some regions was limited, and the UIs were greater in areas with fewer available data. However, the updates of the GBD study will enable methodological improvements with every iteration as well as with the inclusion of the most recent data particularly in data-sparse locations. Second, the GBD estimates were based on a set of data resources such as vital registration, cancer registry, and verbal autopsy. Some low-income countries have no these sources available; thus, their estimates are based on predictive covariates or trends from neighboring countries [49].

## Conclusion

This study indicates that the global burden of cervical cancer has decreased during the past decade; however, it remains high in low-resource settings. There is an urgent need to expand the coverage of HPV vaccination through a national immunization schedule to achieve maximum public health benefit in resource-limited settings.

## Supplementary Information

**Additional file 1: Table S1.** Incidence, DALYs and deaths for cervical cancer in 2017 and percentage changes between 2007 and 2017, by SDI quintiles and by region.

**Additional file 2: Table S2.** Incidence, DALYs and deaths for cervical cancer in 2017 and percentage changes in age-standardized rates between 2007 and 2017, by location.

**Additional file 3: Fig. S1.** Age-specific incidence (A), DALYs (B), and death (C) rates of cervical cancer by SDI quintiles.

## Data Availability

The datasets analyzed for this study are publicly available at https://gbd2017.healthdata.org/gbd-search/.

## Abbreviations

DALYs: Disability-adjusted life-years
SDI: Socio-demographic Index
UIs: Uncertainty intervals
GBD: Global Burden of Disease
GLOBOCAN: Global Cancer Incidence, Mortality and Prevalence
HPV: Human papillomavirus
hrHPV: High-risk types of human papillomavirus
YLDs: Years lived with disability
YLLs: Years of life lost
HICs: High income countries
