# Supplementary material for: Global, regional, and national burden of cervical cancer for 195 countries and territories, 2007–2017: findings from the Global Burden of Disease Study 2017
Source: BMC Womens Health. 2021 Dec 18;21:419. doi: 10.1186/s12905-021-01571-3 (PMC8684284; doi:10.1186/s12905-021-01571-3)
Supplement: Supplementary file 1 — Additional file 1: Table S1. Incidence, DALYs and deaths for cervical cancer in 2017 and percentage changes between 2007 and 2017, by SDI quintiles and by region. [file 12905_2021_1571_MOESM1_ESM.pdf]

Table S1. Incidence, DALYs and deaths for cervical cancer in 2017 and percentage changes between 2007 and 2017, by SDI quintiles and by region.

	Incidence (95%UI)		DALYs (95%UI)		Deaths (95%UI)	
	**2017 counts**	**Percentage changes in cases,2007-2017**	**2017 counts**	**Percentage changes in cases,2007-2017**	**2017 counts**	**Percentage changes in cases,2007-2017**
**Global**	**601186 (554455 to 625402)**	**18.9 (12.8 to 23.0)**	**8061667 (7527014 to 8401647)**	**15.2 (9.5 to 19.2)**	**218631 (204174 to 230958)**	**18.8 (12.9 to 22.8)**
**Low SDI**	**106167 (95862 to 116712)**	**26.4 (17.8 to 35.8)**	**1710473 (1541497 to 1882373)**	**22.2 (14.4 to 31.4)**	**39529(35527 to 43401)**	**24.3(15.9 to 33.3)**
**Low-middle SDI**	**129180 (116270 to 144856)**	**23.6 (16.4 to 32.6)**	**1894281 (1713065 to 2137452)**	**19.1 (12.4 to 28.1)**	**46355(41747 to 53907)**	**22(15.3 to 30.9)**
**Middle SDI**	**186320 (152840 to 195853)**	**23.4 (11.8 to 30.3)**	**2456312 (2009841 to 2581203)**	**18.4 (7.6 to 24.3)**	**64413(58640 to 66525)**	**24.4(13.3 to 30.3)**
**High-middle SDI**	**102547 (89511 to 108141)**	**12.2 (-0.4 to 18.2)**	**1227689 (1073166 to 1290650)**	**6.5 (-6 to 11.8)**	**37849(36968 to 39019)**	**11.3(-1.4 to 16.7)**
**High SDI**	**74634 (72233 to 77053)**	**2.1 (-1.2 to 5.6)**	**742813 (719395 to 767913)**	**-1.7 (-4.6 to 1.2)**	**29748(29499 to 30081)**	**3.2(0.3 to 6.3)**
Central Asia	7101 (6527 to 7760)	4.0 (-4.9 to 13.7)	97090 (90080 to 104919)	-0.6 (-8.2 to 7.5)	2840(2745 to 2944)	0.3(-6.8 to 8.1)
Central Europe	13320 (12602 to 14105)	-15.8 (-20.1 to -11.2)	182097 (173456 to 191669)	-18.9 (-22.8 to -14.8)	7912(7761 to 8071)	-12.8(-16.9 to -8.4)
Eastern Europe	21930 (20770 to 23132)	-18.4 (-23.3 to -13.4)	242922 (233739 to 252335)	-26.2 (-28.8 to -23.3)	10907(10748 to 11084)	-23.5(-26.1to -20.7)
Australasia	1152 (984 to 1344)	8.9 (-7.2 to 29.1)	13238 (11573 to 15127)	7.1 (-7.4 to 23.1)	506(491 to 522)	9.3(-4.2 to 24.6)
High-income Asia Pacific	15490 (14526 to 16613)	-0.1 (-6.7 to 7.0)	121991 (116437 to 128700)	-6.7 (-11 to -1.7)	5174(5095 to 5262)	2.5(-1.8 to 7.5)
High-income North America	23037 (21875 to 24240)	13.4 (7.4 to 20.1)	228627 (217630 to 239893)	13.4 (8.1 to 19.0)	7178(7072 to 7294)	17.5(12.5 to 22.6)
Southern Latin America	10926 (9423 to 12650)	14.3 (-1.7 to 33.4)	121469 (107025 to 137962)	7.1 (-6.4 to 22.2)	4018(3878 to 4170)	7.2(-4.9 to 21)
Western Europe	26183 (24707 to 27712)	-2.4 (-7.7 to 3.5)	260403 (246354 to 274976)	-4.3 (-9.1 to 0.8)	11733(11571 to 11922)	0.3(-4.4 to 5.2)
Andean Latin America	7347 (6216 to 8479)	12.4 (-3.7 to 31.0)	102588 (87071 to 117597)	7.1 (-7.0 to 23.3)	3074(2792 to 3362)	12.8(-1.2 to 28.8)
Caribbean	6705 (5560 to 7824)	13.3 (1.3 to 26.0)	96573 (79167 to 113794)	12.2 (0.9 to 25.2)	2633(2264 to 2989)	15.7(5.1 to 28)
Central Latin America	26756 (25148 to 28660)	16.8 (9.5 to 25.2)	353069 (333387 to 373695)	12.4 (6.2 to 19.6)	9870(9700 to 10057)	15.1(9.0 to 22.2)
Tropical Latin America	24126 (23143 to 25222)	17.6 (12.3 to 23.7)	316023 (305123 to 329010)	11.7 (7.3 to 16.6)	8977(8773 to 9170)	13.6(9.4 to 18.4)
North Africa and Middle East	14577 (12548 to 16180)	20.3 (11.3 to 31.2)	187684 (159194 to 208843)	14.7 (6.8 to 24.4)	4935(4330 to 5410)	17.7(9.8 to 26.9)
East Asia	113036 (73551 to 122613)	42.0 (7.9 to 54.5)	1423547 (923640 to 1544767)	35.1 (1.3 to 46.1)	33281(29153 to 34581)	44.5(9.2 to 56.0)
Oceania	2325 (1583 to 3172)	28.2 (2.9 to 60.4)	32058 (22840 to 42233)	25.9 (5.0 to 53.3)	639(493 to 818)	27.6(8.6 to 51.1)

Southeast Asia	62047 (52363 to 69261)	9.0 (0.6 to 20.4)	806446 (674759 to 906591)	2.9 (-4.4 to 12.9)	23822(19745 to 26867)	7.8(0.5 to 17.1)
Central sub-Saharan Africa	16071 (11496 to 20128)	21.3 (3.8 to 43.2)	265271 (193429 to 332876)	17.0 (-0.5 to 38.5)	6419(4620 to 7788)	18.3(0.7 to 39.4)
Eastern sub-Saharan Africa	39062 (33250 to 46432)	23.6 (13.3 to 36.8)	646308 (549919 to 769675)	18.0 (8.9 to 29.7)	15719(13108 to 18222)	18.7(10.1 to 29.6)
Southern sub-Saharan Africa	12498 (11079 to 13588)	-17.8 (-29.9 to -7.0)	182929 (162744 to 199885)	-17.6 (-27.5 to -7.8)	6582(6057 to 7431)	-10.7(-19.8 to -1.0)
Western sub-Saharan Africa	39955 (31011 to 49933)	30.2 (12.6 to 51.5)	611846 (480371 to 753733)	28.6 (13.7 to 47.7)	13637(10774 to 16838)	29.8(16.2 to 48.4)

UI= 95% uncertainty intervals. DALYs=disability-adjusted life-years. SDI=Socio-demographic Index.
