# Supplementary material for: Global, regional, and national burden of cervical cancer for 195 countries and territories, 2007–2017: findings from the Global Burden of Disease Study 2017
Source: BMC Womens Health. 2021 Dec 18;21:419. doi: 10.1186/s12905-021-01571-3 (PMC8684284; doi:10.1186/s12905-021-01571-3)
Supplement: Supplementary file 2 — Additional file 2: Table S2. Incidence, DALYs and deaths for cervical cancer in 2017 and percentage changes in age-standardized rates between 2007 and 2017, by location. [file 12905_2021_1571_MOESM2_ESM.pdf]

Table S2. Incidence, DALYs and deaths for cervical cancer in 2017 and percentage changes in age-standardized rates between 2007 and 2017, by location.

	**Incidence (95%UI)**			**DALYs(95%UI)**			**Deaths (95%UI)**		
	**2017 counts**	**Percentage changes in cases,2007-2017**	**Percentage changes in age-standardized rates,2007-2017**	**2017 counts**	**Percentage changes in cases,2007-2017**	**Percentage changes in age-standardized rates,2007-2017**	**2017 counts**	**Percentage changes in cases,2007-2017**	**Percentage changes in age-standardized rates,2007-2017**
**Central Asia**	**7101 (6527 to 7760)**	**4.0 (-4.9 to 13.7)**	**-16.2 (-23.2 to -8.6)**	**97090 (90080 to 104919)**	**-0.6 (-8.2 to 7.5)**	**-20.8 (-26.8 to -14.4)**	**2840 (2745 to 2944)**	**0.3 (-6.8 to 8.1)**	**-20.6 (-26 to -14.6)**
Armenia	320 (283 to 360)	-12.5 (-23.3 to 0.8)	-16.0 (-26.8 to -2.5)	4399 (4004 to 4787)	-15.8 (-24.3 to -6.1)	-19.4 (-27.8 to -9.7)	170 (160 to 181)	-9.6 (-18.1 to -0.5)	-15.8 (-23.6 to -6.8)
Azerbaijan	676 (538 to 838)	7.9 (-13.5 to 32.2)	-16.3 (-32.4 to 2.9)	9185 (7523 to 11142)	-0.3 (-17.7 to 19.4)	-24.1 (-36.7 to -9.9)	262 (233 to 294)	2.2 (-14.8 to 21.7)	-24.5 (-36.3 to -10.8)
Georgia	492 (436 to 558)	20.0 (4.0 to 37.6)	27.9 (10.6 to 48.3)	7023 (6366 to 7701)	18.9 (6.23 to 33.20)	25.5 (11.8 to 41.1)	244.4 (223.6 to 267.1)	23.2 (10.4 to 36.9)	27.1 (14.5 to 41.0)
Kazakhstan	1749 (1499 to 2034)	-17.0 (-28.8 to -3.4)	-29.4 (-39.6 to -17.8)	22302 (19977 to 2427)	-24.1 (-32.9 to -14.5)	-35.6 (-43.1 to -27.3)	884 (845 to 925)	-25.2 (-33.1 to -16.7)	-36.8 (-43.2 to -29.3)
Kyrgyzstan	510 (445 to 589)	-2.5 (-16.1 to 14.4)	-20.1 (-30.8 to -6.7)	7238 (6553 to 7966)	-3.3 (-13.3 to 8.3)	-21.6 (-29.4 to - 1goe2.3)	212 (200 to 223)	-0.1 (-9.9 to 10.9)	-18.3 (-26.0 to -9.5)
Mongolia	306 (242 to 384)	16.4 (-8.1 to 49.6)	-20.1 (-35.7 to 1.3)	4658 (3869 to 5695)	14.9 (-7 to 45.3)	-22.8 (-36.7 to -3.1)	117 (102 to 134)	18.5 (-2.0 to 47.6)	-21.5 (-34.6 to -3.8)
Tajikistan	231 (187 to 294)	64.9 (27.6 to 106.9)	14.7 (-9.4 to 41.8)	3432 (2829 to 4321)	60.7 (27.0 to 99.7)	10.5 (-11.2 to 34.6)	61 (53 to 84)	60.3 (29.4 to 95.9)	11.2 (-9.3 to 34.5)
Turkmenistan	465	26.3	2.8	6522	19.9	-4.3	147	24.1	-4.1

	(388 to 546)	(3.5 to 51.3)	(-15.1 to 22.1)	(5618 to 7466)	(1.2 to 40.3)	(-18.3 to 12.3)	(138 to 157)	(6.7 to 44.1)	(-17.4 to 11)
Uzbekistan	2351	16.3	-12.7	32332	12.0	-17.2	789	12.8	-18.1
	(1886 to 2936)	(-8.1 to 45.9)	(-30.1 to 8.8)	(26812 to 39129)	(-9.2 to 38.3)	(-32.5 to 1.6)	(721 to 862)	(-8.1 to 37.8)	(-32.7 to -0.4)
**Central Europe**	**13320**	**-15.8**	**-19.6**	**182097**	**-18.9**	**-23.3**	**7912**	**-12.8**	**-21.1**
	**(12602 to 14105)**	**(-20.1 to -11.2)**	**(-24.1 to -14.8)**	**(173456 to 191669)**	**(-22.8 to -14.8)**	**(-27 to -19.3)**	**(7761 to 8071)**	**(-16.9 to -8.4)**	**(-24.8 to -17.2)**
Albania	127	-6.9	-10.1	1667	-7.6	-14.7	56	0.8	-14.8
	(89 to 172)	(-31.5 to 26.4)	(-34.7 to 21.9)	(1184 to 2224)	(-32.3 to 23.5)	(-37.9 to 14.9)	(48 to 64)	(-25.2 to 33.6)	(-37.1 to 12.9)
Bosnia and	316	-14.2	-8.7	4464	-15.3	-11.7	173	-8.5	-9.8
Herzegovina	(256 to 368)	(-28.4 to 3.4)	(-23.8 to 10.4)	(3759 to 5108)	(-27.4 to -0.3)	(-24 to 4.6)	(148 to 194)	(-20.7 to 6.1)	(-21.4 to 5.0)
Bulgaria	1041	-11.1	-5.4	13521	-15.8	-11.6	544	-14.4	-12.8
	(907 to 1177)	(-22.2 to 1.7)	(-18.4 to 9.3)	(12111 to 15003)	(-24.9 to -6)	(-21.8 to -0.8)	(521 to 567)	(-22.9 to -4.8)	(-22.0 to -3.0)
Croatia	447	-14.1	-17.0	4040	-14.5	-18.7	193	-6.8	-16.0
	(388 to 513)	(-26.4 to -1)	(-29.4 to -3.5)	(3605 to 4525)	(-24.6 to -3.9)	(-28.7 to -8.2)	(183 to 202)	(-17.6 to 4.3)	(-25.5 to -6.1)
Czech Republic	1018	-8.9	-13.1	12114	-12.4	-18.9	543	-8.0	-19.3
	(893 to 1156)	(-20.2 to 4.2)	(-25.1 to 1)	(10879 to 13360)	(-21.6 to -2.5)	(-27.9 to -9.4)	(524 to 564)	(-16.7 to 1.9)	(-27.4 to -10.8)
Hungary	906	-17.2	-20.5	11816	-16.6	-20.6	517	-11.8	-18.4
	(797 to 1029)	(-27.9 to -6.2)	(-31.9 to -8.3)	(10600 to 13059)	(-25.3 to -7.2)	(-29.2 to -11.4)	(499 to 537)	(-20.3 to -2.4)	(-26.4 to -9.6)
Macedonia	183	-2.8	-10.4	2398	-6.6	-15.2	81	-5.6	-17.8
	(151 to 218)	(-17.7 to 17.1)	(-24.8 to 8.1)	(2021 to 2850)	(-20.3 to 12.3)	(-27.6 to 2.1)	(73 to 91)	(-19.7 to 13.3)	(-29.8 to -1.7)
Montenegro	55	-12.5	-17.1	699	-14.4	-20.1	27	-7.5	-16.8
	(45 to 67)	(-28.1 to 6.6)	(-32.8 to 2.5)	(699 to 844)	(-27.6 to 1.7)	(-33.1 to -4.1)	(24 to 31)	(-21.2 to 9.2)	(-29.4 to -2.1)
Poland	3237	-18.8	-26.4	54103	-24.2	-31.0	2629	-17.1	-28.3
	(2908 to 3616)	(-27.9 to -9.5)	(-34.7 to -17.4)	(48535 to 60310)	(-32.5 to -15.3)	(-38.3 to -22.7)	(2543 to 2716)	(-25.4 to -7.9)	(-35.6 to -20.3)
Romania	3810	-17.5	-17.5	50988	-18.2	-19.8	2042	-11.5	-16.6
	(3344 to 4341)	(-27.4 to -6.2)	(-27.9 to -5.6)	(46330 to 55924)	(-26.2 to -9.7)	(-27.9 to -10.8)	(1964 to 2118)	(-19.8 to -2.2)	(-24.7 to -7.8)
Serbia	1352	-19.2	-21.8	17558	-19.7	-22.2	764	-11.8	-17.2

	(1160 to 1564)	(-30.9 to -6.5)	(-33.7 to -7.8)	(15231 to 19924)	(-28.9 to -9.2)	(-31.2 to -11.7)	(696 to 818)	(-21.4 to -1.0)	(-26.1 to -7.0)
Slovakia	682	-2.9	-9.1	7160	-7.4	-15.3	263	-3.5	-15.0
	(523 to 809)	(-17.7 to 14.3)	(-24.1 to 7.5)	(5282 to 8198)	(-20.1 to 6)	(-27.3 to -2.8)	(214 to 281)	(-15.9 to10.0)	(-26.2 to -3.2)
Slovenia	145	-18.0	-23.4	1569	-19.1	-26.2	81	-11.5	-25.2
	(123 to 168)	(-30.7 to -4.3)	(-36.6 to -9.4)	(1371 to 1782)	(-29.7 to -6.3)	(-36 to -14.6)	(76 to 86)	(-22.2 to 1.7)	(-34.4 to -13.8)
**Eastern Europe**	**21930**	**-18.4**	**-21.1**	**242922**	**-26.2**	**-28.3**	**10907**	**-23.5**	**-27.5**
	**(20770 to 23132)**	**(-23.3 to -13.4)**	**(-26.6 to -15.5)**	**(233739 to 252335)**	**(-28.8 to -23.3)**	**(-31 to -25.5)**	**(10748 to 11084)**	**(-26.1to -20.7)**	**(-30.0 to -24.8)**
Belarus	1017	-15.4	-17.3	12554	-22.8	-25.4	568	-20.1	-24.8
	(869 to 1183)	(-27.3 to -1.8)	(-30.1 to -2.4)	(11164 to 14141)	(-31.9 to -13.1)	(-34.4 to -15.5)	(540 to 596)	(-29 to -11)	(-33.0 to -15.9)
Estonia	130	-26.7	-26.9	1471	-30.2	-31.6	80	-23.3	-29.8
	(105 to 159)	(-40.6 to -10.8)	(-42.1 to -9.1)	(1217 to 1769)	(-42.8 to -15.3)	(-44.2 to -16.8)	(75 to 85)	(-36.5 to -7.8)	(-42.3 to -15.2)
Latvia	148	-36.7	-34.5	2015	-40.5	-38.7	134	-34.3	-35.9
	(120 to 180)	(-48.1 to -23.2)	(-47.2 to -19)	(1664 to 2422)	(-50.9 to -28.1)	(-49.9 to -24.6)	(126 to 141)	(-45.3 to -21.3)	(-47.2 to -22.8)
Lithuania	268	-44.4	-42.0	4173	-45.0	-44.1	284	-38.2	-41.5
	(235 to 305)	(-51.2 to -36.2)	(-50.2 to -31.6)	(3709 to 4674)	(-51.3 to -37.4)	(-50.6 to -36.2)	(271 to 297)	(-44.9 to -30)	(-47.8 to -33.6)
Moldova	399	-23.5	-25.9	5317	-27.8	-30.5	223	-23.5	-28.5
	(350 to 455)	(-33.4 to -11.6)	(-36.2 to -13.3)	(4822 to 5838)	(-35.2 to -19.8)	(-37.9 to -22)	(211 to 234)	(-30.9 to -15.6)	(-35.6 to -21.1)
Russia	16048	-11.9	-16.6	164073	-19.9	-23.4	6548	-16.7	-22.1
	(15093 to 17095)	(-18.4 to -5.2)	(-23.4 to -9.1)	(158658 to 169649)	(-22.7 to -17.1)	(-26.2 to -20.6)	(6431 to 6685)	(-19.3 to -14)	(-24.6 to -19.5)
Ukraine	3920	-35.3	-35.1	53319	-39	-39	3070	-36.7	-38.0
	(3458 to 4394)	(-42.7 to -26.2)	(-43.0 to -25.1)	(48559 to 58892)	(-44.8 to -31.8)	(-44.9 to -31.7)	(2956 to 3186)	(-42.2 to -29.9)	(-43.4 to -31.0)
High income	78737	1.4	-7.4	742813	2.1	-7.0	28609	6.1	-8.3
	(76288 to 81254)	(-1.8 to 4.7)	(-10.5 to -4.2)	(719395 to 767913)	(-1.7 to 5.9)	(-10.6 to -3.3)	(28327 to 28966)	(2.5 to 9.8)	(-11.6 to -5.0)
**Australasia**	**1152**	**8.9**	**-6.0**	**13238**	**7.1**	**-10.7**	**506**	**9.3**	**-11.8**
	**(984 to 1344)**	**(-7.2 to 29.1)**	**(-21.5 to 12.7)**	**(11573 to 15127)**	**(-7.4 to 23.1)**	**(-22.8 to 2.8)**	**(491 to 522)**	**(-4.2 to 24.6)**	**(-23.2 to 0.8)**
Australia	968	13.0	-3.7	11186	9.9	-8.9	424	11.2	-10.5

	(801 to 1156)	(-6.8 to 36.9)	(-21.8 to 18.9)	(9460 to 13114)	(-7.5 to 29.2)	(-23.6 to 7.5)	(409 to 439)	(-4.9 to 29.2)	(-23.9 to 4.6)
New Zealand	185	-8.4	-15.0	2052	-6.0	-18.7	82	-0.3	-18.5
	(163 to 212)	(-21.2 to 6.0)	(-28.4 to 0.2)	(1864 to 2267)	(-15.3 to 4.2)	(-27 to -9.5)	(78 to 86)	(-9.5 to 10)	(-26.2 to -9.9)
**High-income Asia Pacific**	**15490**	**-0.1**	**-7.4**	**121991**	**-6.7**	**-15.2**	**5174**	**2.5**	**-15.8**
	**(14526 to 16613)**	**(-6.7 to 7.0)**	**(-14.3 to 0.4)**	**(116437 to 128700)**	**(-11 to -1.7)**	**(-19.4 to -10.6)**	**(5095 to 5262)**	**(-1.8 to 7.5)**	**(-19.6 to -11.3)**
Brunei	68	33.0	0.3	627	31.8	-4.5	13	37.4	-5.3
	(53 to 85)	(4.7 to 69.8)	(-19.2 to 24.8)	(520 to 752)	(7.5 to 60.2)	(-20.8 to 14.5)	(11 to 15)	(15 to 63.8)	(-20 to 11.4)
Japan	11866	2.7	-1.3	90563	-4.7	-9.2	3868	5.3	-9.3
	(11020 to 12806)	(-4.7 to 11.2)	(-10.0 to 8.8)	(86462 to 95764)	(-9.2 to 0.6)	(-13.4 to -3.9)	(3800 to 3946)	(0.8 to 10.5)	(-13.5 to -4.4)
Singapore	268	3.7	-23.2	2268	-2.8	-30.0	77	2.1	-32.2
	(229 to 314)	(-11.9 to 21.6)	(-34.5 to -10.6)	(2015 to 2575)	(-14.7 to 10.1)	(-38.4 to -20.3)	(73 to 82)	(-9.4 to 15.2)	(-39.7 to -23.5)
South Korea	3288	-9.8	-24.2	28533	-13.2	-31.4	1216	-6.8	-34.3
	(2825 to 3829)	(-22.9 to 5.0)	(-35.9 to -11.3)	(25518 to 31784)	(-22.8 to -3.0)	(-39.1 to -23.1)	(1177 to 1256)	(-16.4 to 3.5)	(-40.9 to -26.9)
**High-income North America**	**23037**	**13.4**	**2.2**	**228627**	**13.4**	**0.2**	**7178**	**17.5**	**-0.1**
	**(21875 to 24240)**	**(7.4 to 20.1)**	**(-3.8 to 8.7)**	**(217630 to 239893)**	**(8.1 to 19.0)**	**(-4.7 to 5.6)**	**(7072 to 7294)**	**(12.5 to 22.6)**	**(-4.5 to 4.5)**
Canada	2275	7.8	-3.0	22590	5.4	-8.4	874	9.5	-10.7
	(1961 to 2611)	(-7.0 to 23.8)	(-17.9 to 13.5)	(20296 to 25241)	(-6.1 to 18.5)	(-18.9 to 3.1)	(841 to 908)	(-0.9 to 21.9)	(-19.8 to -0.2)
Greenland	7	-24.4	-26.6	83	-26.2	-31.3	3	-18.7	-29.6
	(6 to 9)	(-39.4 to -5.0)	(-40.6 to -7.6)	(69 to 104)	(-39.3 to -9.1)	(-43.2 to -16.0)	(3 to 4)	(-32.1 to -1.3)	(-40.8 to -15.5)
USA	20755	14.1	2.8	205951	14.4	1.3.0	6301	18.6	1.2
	(19628 to 21918)	(7.5 to 21.1)	(-3.5 to 9.9)	(195837 to 216547)	(8.5 to 20.4)	(-4.3 to 7.0)	(6206 to 6407)	(13.2 to 24.1)	(-3.7 to 6.1)
**Southern Latin America**	**10926**	**14.3**	**-2.1**	**121469**	**7.1**	**-9.6**	**4018**	**7.2**	**-11.5**
	**(9423 to 12650)**	**(-1.7 to 33.4)**	**(-16.2 to 14.9)**	**(107025 to 137962)**	**(-6.4 to 22.2)**	**(-21.1 to 3.2)**	**(3878 to 4170)**	**(-4.9 to 21)**	**(-21.8 to 0.3)**
Argentina	7983	18.4	0.4	88896	9.8	-7.2	2807	7.9	-8.9
	(6549 to 9618)	(-3.4 to 44.5)	(-18.5 to 23.4)	(73917 to 104463)	(-8.6 to 29.8)	(-22.9 to 10.1)	(2679 to 2949)	(-8.8 to 26.1)	(-23.3 to 6.7)
Chile	2302	5.9	-11.1	25173	2.0	-18.0	898	8.0	-19.2

	(1885 to 2809)	(-13.5 to 28)	(-28.1 to 7.9)	(21051 to 30076)	(-14.9 to 21.5)	(-31.6 to -2.3)	(860 to 934)	(-8.9 to 27.6)	(-31.9 to -4.4)
Uruguay	640	0.5	-6.2	7394	-4.2	-12.0	313	-1.6	-12.7
	(522 to 768)	(-18.3 to 20.9)	(-24.9 to 14.4)	(6184 to 8627)	(-20.4 to 12.8)	(-27.3 to 4.2)	(296 to 331)	(-17 to 14.7)	(-26.9 to 2.1)
**Western Europe**	**26183**	**-2.4**	**-7.3**	**260403**	**-4.3**	**-11.9**	**11733**	**0.3**	**-11.8**
	**(24707 to 27712)**	**(-7.7 to 3.5)**	**(-12.4 to -0.9)**	**(246354 to 274976)**	**(-9.1 to 0.8)**	**(-16.3 to -7.2)**	**(11571 to 11922)**	**(-4.4 to 5.2)**	**(-16.1 to -7.3)**
Andorra	4	-4.2	-2.8	38	9.4	1.6	1.0	22.9	6.6
	(3 to 6)	(-26.3 to 26.4)	(-25 to 30.1)	(30 to 49)	(-12.2 to 39.0)	(-18.3 to 29.6)	(1 to 2)	(-2.3 to 54.7)	(-14.6 to 35.5)
Austria	558	-6.6	-12.2	5485	-8.1	-16.4	259	-4.9	-16.3
	(494 to 631)	(-17.9 to 6.5)	(-24.1 to 2.5)	(4943 to 6077)	(-17.7 to 2.7)	(-25.6 to -6.5)	(247 to 272)	(-14.4 to 5.8)	(-25 to -6.6)
Belgium	613	-13.5	-20.6	6503	-14.2	-22.2	330	-7.4	-19.6
	(534 to 700)	(-25.1 to 0.3)	(-32.8 to -6.5)	(5782 to 7281)	(-23.7 to -3.4)	(-31.5 to -11.9)	(317 to 344)	(-17.1 to 3.5)	(-28.2 to -9.9)
Cyprus	57	10.8	-16.8	567	5.0	-22.0	20	7.9	-23.1
	(46 to 69)	(-9.9 to 36.5)	(-32.5 to 2.9)	(440 to 670)	(-11.8 to 24.3)	(-34.4 to -7.7)	(16 to 23)	(-9.8 to 27.1)	(-35.6 to -9.2)
Denmark	380	-14.2	-17.2	3883	-15.6	-23.0.	202	-11.7	-22.2
	(332 to 434)	(-25 to -0.7)	(-29.4 to -1.5)	(3483 to 4346)	(-25.1 to -4.5)	(-31.9 to -11.9)	(193 to 211)	(-21.1 to -1.1)	(-30.6 to -12.6)
Finland	213	-2.0	-12.2	2167	-1	-13.2	102	7.7	-9.4
	(186 to 242)	(-15.9 to 13.2)	(-25.9 to 3.1)	(1927 to 2428)	(-12.9 to 12.2)	(-23.9 to -1.2)	(96 to 108)	(-5.1 to 21.3)	(-20.0 to 2.5)
France	3749	-4.8	-11.6	39419	-5.6	-14.0	1850	1.2	-13.9
	(3249 to 4283)	(-17.5 to 9.9)	(-25.1 to 4.5)	(35150 to 44082)	(-16.2 to 6.0)	(-24.0 to -2.8)	(1779 to 1927)	(-9.3 to 11.7)	(-23.1 to -3.7)
Germany	5662	2.9	1.6	58000	-3.2	-7.2	2544	1.9	-6.4
	(4710 to 6761)	(-13.3 to 23.0)	(-16.6 to 24.4)	(48697 to 68399)	(-18.2 to 14.8)	(-21.8 to 10.6)	(2468 to 2624)	(-13.3 to 19)	(-20.7 to 10.2)
Greece	702	0.1	0.8	7525	1.0	-0.5	325	3.6	-7.3
	(598 to 806)	(-14.6 to 15.0)	(-16 to 18.2)	(6641 to 8402)	(-10.9 to 13.9)	(-12.8 to 12.9)	(309 to 342)	(-7.9 to 16.4)	(-17.7 to 4.2)
Iceland	12	4.8	-8.8	122	3.9	-12.5	5	8.8	-12.7
	(11 to 14)	(-10.8 to 23.0)	(-23.6 to 8.5)	(110 to 136)	(-8.9 to 18.7)	(-23.9 to 0.6)	(4 to 5)	(-3.8 to 22)	(-22.6 to -1.5)
Ireland	304	1.3	-14	2811	-0.6	-18.7	98	3.2	-17.9

	(255 to 360)	(-14.8 to 22.2)	(-28.3 to 5.1)	(2482 to 3188)	(-12.8 to 14.2)	(-28.7 to -6.7)	(93 to 104)	(-8.4 to 17.7)	(-27.2 to -6.2)
Israel	346	12.7	-10.2	3560	6.9	-15.9	134	10.7	-16.9
	(296 to 407)	(-3.9 to 31.3)	(-24.6 to 5.0)	(3158 to 3985)	(-5.4 to 22.6)	(-25.6 to -3.2)	(127 to 142)	(-1.2 to 24.4)	(-25.8 to -6.5)
Italy	3118	-0.4	-4.9	33146	-0.7	-8.5	1577	2.9	-9.8
	(2708 to 3576)	(-13.9 to 14.1)	(-19.2 to 11.4)	(29399 to 36958)	(-12 to 11.1)	(-19.1 to 3.7)	(1517 to 1641)	(-8.0 to 14.1)	(-19.7 to 0.6)
Luxembourg	28	6.0	-15.2	270	4.3	-16.9	11	7.6	-15.2
	(23 to 33)	(-14.8 to 30.0)	(-32.9 to 5.8)	(225 to 319)	(-13.9 to 24.6)	(-31.6 to 0.1)	(10 to 12)	(-10.7 to 30.0)	(-29.9 to 2.3)
Malta	18	9.5	-3.0	201	6.2	-8.3	8	15.3	-8.2
	(16 to 21)	(-5.7 to 27.3)	(-18.4 to 14.6)	(178 to 225)	(-6.6 to 20.9)	(-19.8 to 5.1)	(7 to 8)	(2.0 to 31.5)	(-18.5 to 4.6)
Netherlands	882	2.2	-1.6	8738	3.4	-6.0	370	9.1	-6.3
	(772 to 1017)	(-12.1 to 17.5)	(-17.6 to 16.7)	(7841 to 9673)	(-7.6 to 15.1)	(-16.4 to 5.3)	(355 to 389)	(-1.9 to 20.5)	(-15.5 to 3.6)
Norway	280	-13.8	-23.6	2601	-14.9	-26.7	129	-10.7	-22.9
	(257 to 305)	(-21.1 to -5.2)	(-31.1 to -14.9)	(2465 to 2752)	(-19.2 to -10.0)	(-30.5 to -22.2)	(126 to 133)	(-15 to -5.9)	(-26.8 to -18.8)
Portugal	941	-20.7	-28.8	8718	-18.4	-28.3	416	-9.1	-24.8
	(817 to 1080)	(-31.7 to -8.4)	(-39.8 to -16.6)	(7691 to 9766)	(-28.2 to -7.0)	(-37.8 to -18.3)	(395 to 435)	(-19.2 to 1.9)	(-33.5 to -14.9)
Spain	2820	-5.7	-15.2	25451	-3	-15.4	1080	3.7	-12.8
	(2453 to 3224)	(-17.8 to 8.7)	(-27.4 to -1.6)	(22988 to 28301)	(-13.2 to 8.2)	(-24.5 to -5.0)	(1042 to 1121)	(-5.9 to 15.1)	(-21.4 to -3.4)
Sweden	548	-4.2	-8.5	5652	-6.5	-14.1	306	-8.4	-15.5
	(492 to 608)	(-14.1 to 7.9)	(-20 to 5.5)	(5168 to 6214)	(-15.4 to 2.8)	(-22.3 to -4.4)	(292 to 320)	(-16.7 to 0)	(-23.4 to -7.6)
Switzerland	370	-15.4	-25.5	3908	-16.2	-28	218	-11.8	-26.5
	(324 to 424)	(-27.2 to -3.2)	(-37.3 to -13.2)	(3500 to 4365)	(-26.1 to -6.2)	(-36.9 to -18.7)	(209 to 228)	(-21.4 to -1.2)	(-35.1 to -17.5)
UK	4552	2.0	-4.0	41367	-2.9	-9.7	1736	-2.2	-11.0
	(4377 to 4759)	(-2.1 to 6.4)	(-8.5 to 0.8)	(39924 to 42986)	(-5.8 to 0.4)	(-12.5 to -6.5)	(1707 to 1772)	(-4.9 to 1.1)	(-13.6 to -8.0)
England	3770	1.8	-4.6	34105	-2.8	-10.0	1442	-2.2	-11.1
	(3611 to 3932)	(-2.5 to 6.2)	(-9.2 to 0.4)	(32981 to 35231)	(-5.5 to 0.3)	(-12.6 to -6.9)	(1414 to 1474)	(-5.0 to 0.9)	(-13.5 to -8.3)
Northern	122	-2.9	-11.0	1117	-6.4	-16.1	46	-2.8	-15.9

Ireland	(101 to 146)	(-18.8 to 18.5)	(-27.1 to 11.8)	(954 to 1305)	(-20.2 to 10.5)	(-28.7 to -0.1)	(43 to 49)	(-17 to 12)	(-28.2 to -1.8)
Scotland	438	12.2	10.1	4038	2.9	-0.1	150	3.8	-3.5
	(377 to 511)	(-3.9 to 31.7)	(-8.2 to 32.5)	(3540 to 4579)	(-9.9 to 17.2)	(-13.7 to 15)	(143 to 157)	(-8.4 to 17)	(-15.1 to 9.8)
Wales	221	-9.0	-12.6	2107	-12.4	-16.7	97	-9.7	-17.0
	(191 to 259)	(-23.3 to 7.1)	(-28.8 to 6.6)	(1864 to 2336)	(-23.1 to -0.5)	(-27.8 to -4.3)	(92 to 104)	(-20 to 1.6)	(-26.8 to -6.2)
**Andean Latin**	**7347**	**12.4**	**-15.6**	**102588**	**7.1**	**-19.8**	**3074**	**12.8**	**-18.0**
**America**	**(6216 to 8479)**	**(-3.7 to 31.0)**	**(-27.5 to -1.7)**	**(87071 to 117597)**	**(-7 to 23.3)**	**(-30.3 to -7.7)**	**(2792 to 3362)**	**(-1.2 to 28.8)**	**(-28.1 to -6.4)**
Bolivia	1699	14.3	-14.9	26103	9.7	-18.5	760	14.5	-16.3
	(1180 to 2253)	(-12.2 to 47.4)	(-34.1 to 8.7)	(18479 to 34552)	(-15 to 38.2)	(-36.7 to 2.2)	(597 to 953)	(-10.2 to 42.0)	(-34.0 to 3.4)
Ecuador	1946	16.3	-12.4	26497	11.3	-16.2	783	17.3	-15.0
	(1632 to 2281)	(-3.3 to 41.0)	(-27.1 to 5.6)	(22808 to 30794)	(-5.9 to 31.6)	(-29 to -1.0)	(724 to 847)	(0.9 to 36.8)	(-26.7 to -0.9)
Peru	3702	9.7	-17.6	49987	3.7	-22.3	1531	9.6	-20.6
	(2809 to 4669)	(-15.1 to 39.6)	(-36.1 to 4.9)	(38857 to 62097)	(-19.7 to 30.7)	(-39.6 to -2.3)	(1314 to 1728)	(-13.8 to 36.9)	(-37.2 to -1.1)
**Caribbean**	**6705**	**13.3**	**-2.4**	**96573**	**12.2**	**-4.9**	**2633**	**15.7**	**-5.3**
	**(5560 to 7824)**	**(1.3 to 26)**	**(-12.8 to 8.6)**	**(79167 to 113794)**	**(0.9 to 25.2)**	**(-14.6 to 6.3)**	**(2264 to 2989)**	**(5.1 to 28)**	**(-13.8 to 4.8)**
Antigua and	11	21.3	4.3	147	21.4	0.8	4	24.7	-0.1
Barbuda	(10 to 13)	(2.1 to 46.2)	(-12.3 to 25.1)	(129 to 166)	(5.3 to 40.7)	(-12.6 to 17.0)	(4 to 4)	(10.0 to 43.7)	(-11.7 to 15)
The Bahamas	63	24.4	4.3	796	26.8	2.3	18	31.8	1.9
	(52 to 76)	(2.4 to 52.3)	(-14 to 27.3)	(676 to 931)	(6.7 to 49.0)	(-13.6 to 20.0)	(17 to 20)	(12.5 to 54.1)	(-13.5 to 18.7)
Barbados	59	10.1	-5.6	765	13.2	-5.3	24	21.4	-1.2
	(50 to 69)	(-8.5 to 31.7)	(-22.2 to 14)	(653 to 878)	(-3.9 to 32.1)	(-20 to 11.0)	(22 to 26)	(4.8 to 40.5)	(-14.9 to 14.4)
Belize	59	30.2	-14.7	799	28.2	-17.3	17	30.2	-16.7
	(52 to 69)	(11 to 54.7)	(-26.2 to -0.5)	(714 to 896)	(13.4 to 46.0)	(-26.4 to -6.0)	(16 to 19)	(16.2 to 46.7)	(-25.3 to -6.6)
Bermuda	4	-15.5	-24.9	51	-17.8	-28.9	2	-7.6	-28.3
	(4 to 5)	(-29.8 to 0.7)	(-38.6 to -9.5)	(44 to 60)	(-30.9 to -3.0)	(-40.4 to -16.2)	(2 to 3)	(-21.2 to 8.3)	(-38.6 to -15.9)
Cuba	1507	-7.3	-11.6	19208	-5.7	-14.7	643	4.1	-11.8

	(1248 to 1806)	(-23.5 to 11.6)	(-27.4 to 7.7)	(16098 to 22730)	(-21.1 to 12.6)	(-29 to 1.9)	(615 to 673)	(-12.5 to 21.7)	(-25.8 to 3.6)
Dominica	14	10.6	3.5	185	10.2	1.6	6	12.4	1.7
	(12 to 16)	(-6.8 to 30.2)	(-13.3 to 23.3)	(163 to 209)	(-4.9 to 25.8)	(-13 to 16.7)	(6 to 7)	(-1.5 to 27.2)	(-11.4 to 15.2)
Dominican	1208	26.9	2.8	15959	23.2	-1.7	411	25.6	-2.5
Republic	(943 to 1538)	(-4.0 to 69.0)	(-21.7 to 35.4)	(12428 to 20098)	(-5.6 to 58.8)	(-24.6 to 26.1)	(352 to 484)	(-2.6 to 59.3)	(-24.3 to 23.4)
Grenada	20	9.3	-2.1	274	9.8	-3.3	8	23.7	-1.7
	(17 to 22)	(-5.4 to 26.7)	(-15.7 to 15.0)	(246 to 304)	(-3.4 to 24.7)	(-15.2 to 10.1)	(8 to 9)	(9.7 to 40.0)	(-12.9 to 10.9)
Guyana	131	8.5	-1.8	1970	5.2	-6.8	54	10.6	-6.2
	(104 to 162)	(-13.8 to 35.5)	(-21.4 to 21.6)	(1606 to 2363)	(-14.0 to 28.1)	(-23.6 to 12.6)	(50 to 58)	(-8.1 to 32.9)	(-22.1 to 12.2)
Haiti	2257	22.6	-11.4	37940	17.4	-14.6	903	17.7	-13.6
	(1362 to 3177)	(-3.2 to 53.5)	(-29.0 to 9.2)	(23427 to 52689)	(-6.2 to 44.5)	(-31.2 to 5.0)	(562 to 1221)	(-4.8 to 44.7)	(-29.0 to 5.0)
Jamaica	469	28.4	12.3	6420	28.1	10.2	171	26.4	7.8
	(363 to 601)	(-0.1 to 67.4)	(-12.6 to 46.6)	(5068 to 8114)	(0.4 to 64.0)	(-13.3 to 41.0)	(161 to 183)	(1.2 to 58.4)	(-14.1 to 35.0)
Puerto Rico	292	9.5	6.5	3407	8.4	2	118	14.5	-1.0
	(252 to 336)	(-5.5 to 27.4)	(-10.2 to 26.6)	(3021 to 3840)	(-4.4 to 23.9)	(-10.4 to 17.2)	(111 to 125)	(1.7 to 29.4)	(-12.4 to 12.1)
Saint Lucia	31	25.9	0.1	431	25.9	-2.9	11	29.8	-2.0
	(27 to 36)	(4.9 to 48.0)	(-16.3 to 17.2)	(379 to 489)	(8.2 to 45.1)	(-16.6 to 12.4)	(10 to 12)	(13.4 to 47.1)	(-14.3 to 11.2)
Saint Vincent and	28	18.0	2.3	389	21.1	1.8	10	24.1	1.9
the Grenadines	(24 to 33)	(-0.9 to 39.5)	(-14.5 to 21.2)	(343 to 441)	(4.9 to 38.7)	(-12 to 16.8)	(9 to 11)	(8.9 to 39.8)	(-10.8 to 15.0)
Suriname	102	16.9	-4.6	1449	12.4	-10.7	39	18.2	-11.0
	(85 to 121)	(-4.8 to 42.2)	(-22.4 to 15.8)	(1241 to 1702)	(-7.5 to 34.2)	(-25.7 to 6.1)	(35 to 43)	(-0.8 to 38.9)	(-25.0 to 4.2)
Trinidad and	195	3.1	-13.9	2718	1.1	-17	92	6.9	-17.3
Tobago	(141 to 262)	(-26.2 to 41.2)	(-38.8 to 18.2)	(2012 to 3593)	(-26.2 to 35.7)	(-39.4 to 11.8)	(87 to 98)	(-19.8 to 40.6)	(-38.1 to 8.7)
Virgin Islands	15	2.7	-2.2	194	4.2	-3.2	6	14.9	-3.2
	(12 to 18)	(-15.2 to 23.9)	(-21.1 to 21.3)	(162 to 230)	(-11.7 to 23.6)	(-19.3 to 16.8)	(6 to 7)	(-1.7 to 34.0)	(-17.1 to 14.1)
**Central Latin**	**26756**	**16.8**	**-11.7**	**353069**	**12.4**	**-16.5**	**9870**	**15.1**	**-18.4**

**America**	**(25148 to 28660)**	**(9.5 to 25.2)**	**(-17 to -5.6)**	**(333387 to 373695)**	**(6.2 to 19.6)**	**(-21.1 to -11.2)**	**(9700 to 10057)**	**(9.0 to 22.2)**	**(-22.6 to -13.6)**
Colombia	4813	5.4	-18.8	62111	0.1	-24.4	1981	5.5	-26.5
	(3970 to 5835)	(-13.3 to 27.7)	(-33.2 to -1.5)	(52085 to 73451)	(-15.5 to 19)	(-36.3 to -10.0)	(1909 to 2054)	(-10.6 to 23.2)	(-37.5 to -13.9)
Costa Rica	448	48.4	15.2	5491	41.2	8.2	128	47.5	6.4
	(387 to 521)	(25.5 to 75.8)	(-2.3 to 35.7)	(4880 to 6161)	(22.4 to 62.1)	(-6.2 to 24.3)	(121 to 137)	(28.3 to 68.1)	(-7.4 to 21.7)
El Salvador	971	-2.3	-18.8	13188	-4.7	-22	454	-0.8	-20.7
	(709 to 1282)	(-27.4 to 28.8)	(-39.7 to 6.8)	(9897 to 17063)	(-26.8 to 24.4)	(-40.2 to 1.8)	(370 to 497)	(-23.1 to 27.8)	(-38.6 to 2.5)
Guatemala	1901	30.8	-11.0	28092	26.5	-13.4	668	29.4	-13.7
	(1558 to 2287)	(8.5 to 57.2)	(-25.2 to 6.5)	(23537 to 33176)	(6.1 to 50.3)	(-27.0 to 2.7)	(635 to 706)	(9.8 to 51.9)	(-26.4 to 1.0)
Honduras	367	32.8	-7.9	4917	29.6	-10.7	109	36.9	-6.8
	(237 to 670)	(-8.7 to 85.4)	(-36.0 to 26.8)	(3252 to 9156)	(-7.5 to 79.5)	(-36.0 to 21.8)	(75 to 188)	(-2.3 to 84.7)	(-32.6 to 24.5)
Mexico	11896	17.1	-11.7	159023	12.7	-16.7	4574	13.6	-19.3
	(11425 to 12413)	(12.0 to 22.5)	(-15.5 to -7.7)	(153369 to 165325)	(8.5 to 17.4)	(-19.8 to -13.2)	(4484 to 4654)	(9.4 to 18.2)	(-22.3 to -16.1)
Nicaragua	767	8.3	-20.6	10340	5.1	-23.9	290	10.8	-21.9
	(620 to 961)	(-15 to 37.3)	(-37.1 to 0.0)	(8432 to 12499)	(-15.2 to 29.4)	(-38.2 to -6.6)	(255 to 329)	(-9.4 to 33.9)	(-36.1 to -6.3)
Panama	478	25.9	-2.3	5944	18.2	-10.3	161	20.1	-13.3
	(407 to 554)	(6.4 to 48.7)	(-17.3 to 15.2)	(5191 to 6747)	(1.9 to 35)	(-22.5 to 2.7)	(153 to 168)	(5.0 to 35.9)	(-24.3 to -2.0)
Venezuela	5114	25.5	-4.3	63963	21.9	-8.3	1505	26.9	-8.2
	(3872 to 6583)	(-3.6 to 61.2)	(-25.6 to 22.1)	(50547 to 79094)	(-3.8 to 51.9)	(-27.5 to 14.4)	(1447 to 1566)	(1.5 to 57.2)	(-26.4 to 13.4)
**Tropical Latin**	**24126**	**17.6**	**-8.1**	**316023**	**11.7**	**-14.1**	**8977**	**13.6**	**-16.5**
**America**	**(23143 to 25222)**	**(12.3 to 23.7)**	**(-12.2 to -3.3)**	**(305123 to 329010)**	**(7.3 to 16.6)**	**(-17.5 to -10.3)**	**(8773 to 9170)**	**(9.4 to 18.4)**	**(-19.6 to -13.0)**
Brazil	23005	17.9	-7.7	300598	11.9	-13.9	8543	13.8	-16.5
	(22072 to 23992)	(12.6 to 23.7)	(-11.8 to -3.3)	(290268 to 312418)	(7.7 to 16.7)	(-17.1 to -10.2)	(8349 to 8735)	(9.6 to 18.6)	(-19.5 to -13)
Paraguay	1121	12.2	-15.1	15425	7.8	-18.5	434	10.3	-17.3
	(794 to 1485)	(-19.5 to 47.4)	(-39.0 to 10.9)	(11161 to 19986)	(-21.3 to 41.5)	(-40.4 to 6.4)	(363 to 486)	(-17.8 to 42.1)	(-38.0 to 6.0)
**North Africa and**	**14577**	**20.3**	**-13.2**	**187684**	**14.7**	**-17.9**	**4935**	**17.7**	**-16.8**

**Middle East**	**(12548 to 16180)**	**(11.3 to 31.2)**	**(-19.3 to -5.9)**	**(159194 to 208843)**	**(6.8 to 24.4)**	**(-23.5 to -11.4)**	**(4330 to 5410)**	**(9.8 to 26.9)**	**(-22.3 to -10.5)**
Afghanistan	1490	22.6	-11.2	24265	20.0	-16.8	536	17.7	-15.3
	(648 to 2300)	(-6.5 to 66.5)	(-30.5 to 15.8)	(10394 to 36592)	(-5.4 to 58.7)	(-32.1 to 6.3)	(204 to 803)	(-4.1 to 50.1)	(-29.3 to 5)
Algeria	1923	21.0	-13.6	23489	13.9	-19.4	615	15.4	-19.1
	(1534 to 2336)	(-2.1 to 49.2)	(-29.1 to 5.2)	(19482 to 27485)	(-3.7 to 36.0)	(-31.5 to -4.5)	(523 to 701)	(-1.4 to 37.1)	(-30.8 to -4.8)
Bahrain	22	45.5	-19.1	261	43.7	-24.3	5.0	50.0	-23.4
	(18 to 27)	(16.0 to 79.4)	(-33.7 to -1.8)	(219 to 318)	(18.6 to 75.0)	(-36.6 to -9.0)	(5 to 7)	(24.9 to 81.2)	(-36.1 to -8.3)
Egypt	1192	29.5	-0.5	15065	21.8	-6.1	357	25.7	-3.9
	(920 to 1543)	(1.8 to 68.8)	(-20.5 to 26.3)	(12003 to 18768)	(-2.0 to 50.5)	(-23.6 to 14.2)	(316 to 406)	(3.4 to 52.2)	(-19.8 to 15.2)
Iran	1839	32.9	-1.8	20993	33.1	-3.9	507	43.2	1.0
	(1466 to 2076)	(17.2 to 49.7)	(-12.2 to 8.9)	(16868 to 22533)	(21.8 to 42.7)	(-12.6 to 2.6)	(439 to 531)	(29.3 to 53)	(-9.6 to 8.0)
Iraq	416	1.1	-34.6	5636	-1.9	-37.3	164	2.5	-36.1
	(336 to 512)	(-30.8 to 47.7)	(-54.1 to -7.1)	(4670 to 6812)	(-31.5 to 40.2)	(-55.7 to -11.9)	(130 to 223)	(-27.4 to 42.5)	(-54.1 to -13.2)
Jordan	125	18.6	-36.2	1482	14.2	-40.5	39	22.4	-36.9
	(94 to 168)	(-9.2 to 60.6)	(-50.8 to -14.2)	(1139 to 1961)	(-12.2 to 50.6)	(-53.9 to -20.8)	(34 to 45)	(-5.3 to 64.1)	(-50.9 to -16.3)
Kuwait	54	28.4	-44.7	515	18.4	-48.7	12	14.7	-48.2
	(44 to 67)	(3.2 to 58.9)	(-53.8 to -33.8)	(447 to 594)	(1.2 to 39.5)	(-55.5 to -40.7)	(11 to 13)	(-0.1 to 33.2)	(-54.6 to -40.3)
Greenland	253	36.0	-8.4	2505	24.5	-15.4	64	-18.7	-12.4
	(201 to 318)	(9.3 to 69.7)	(-25.7 to 12.3)	(2088 to 3047)	(3 to 48.9)	(-29.6 to 1)	(56 to 73)	(-32.1 to -1.3)	(-26.1 to 3.2)
Libya	334	62.1	8.6	3759	55.2	0.6	69	53.8	1.6
	(232 to 453)	(16 to 112.6)	(-18.9 to 40.9)	(2740 to 4858)	(16.7 to 98.8)	(-22.5 to 28.3)	(57 to 83)	(17.7 to 95.8)	(-21 to 30.1)
Morocco	2272	11.6	-13.2	31984	5.3	-19.5	923	9.5	-18
	(1640 to 3085)	(-12.8 to 43.5)	(-31.5 to 10.7)	(23145 to 42220)	(-16.3 to 33)	(-35.8 to 0.5)	(721 to 1099)	(-12.5 to 38.7)	(-34.2 to 3.5)
Oman	77	39.5	-20.8	790	30.4	-24.4	17	34.6	-20.2
	(55 to 102)	(3.3 to 87.2)	(-38.6 to -0.2)	(595 to 1005)	(2.2 to 66.2)	(-39.3 to -6.6)	(13 to 22)	(9 to 64.5)	(-33.6 to -4.8)
Palestine	63	27.1	-14.4	825	24.2	-16.9	23	21.9	-17.3

	(47 to 77)	(2.4 to 64.2)	(-29.9 to 9.9)	(624 to 981)	(2.4 to 58)	(-31.4 to 5.7)	(19 to 26)	(1.1 to 55.1)	(-31.4 to 5.7)
Qatar	23	97.6	-22.6	228	77.8	-27.9	4	74.5	-29
	(16 to 30)	(50.3 to 154.0)	(-38.8 to -4.0)	(172 to 297)	(41 to 121.6)	(-42.2 to -10.7)	(3 to 5)	(39.9 to 115.9)	(-41.9 to -12.2)
Saudi Arabia	434	69.2	-3.1	4348	43.0	-14.5	90	30.4	-16.5
	(312 to 609)	(25.6 to 131.0)	(-22.7 to 25.1)	(3293 to 5837)	(10.6 to 86.4)	(-30.8 to 5.8)	(77 to 105)	(3.7 to 64.0)	(-31.3 to 1.5)
Sudan	697	20.3	-12.9	9644	13.0	-17.8	251	11.7	-16.7
	(415 to 1008)	(-8.8 to 57.9)	(-31.6 to 11.5)	(5691 to 13807)	(-12.2 to 44.6)	(-34.9 to 4.8)	(148 to 363)	(-10.2 to 40.9)	(-32.8 to 4.9)
Syria	243	9.2	-4.8	3051	9.9	-10.8	83	17.8	-10.2
	(175 to 330)	(-22.2 to 47.6)	(-30.4 to 25.8)	(2231 to 3925)	(-20 to 44.0)	(-33.6 to 14.9)	(70 to 97)	(-10.8 to 49.7)	(-31.1 to 12.1)
Tunisia	365	9.6	-14.8	4075	9.1	-18.2	125	16.1	-16.1
	(252 to 499)	(-20.6 to 47.8)	(-38.1 to 14.2)	(2912 to 5434)	(-19.3 to 43.3)	(-39.5 to 7.6)	(97 to 161)	(-13.3 to 50.1)	(-36.8 to 8.4)
Turkey	1877	-1.4	-23.9	22811	-5.1	-28.3	801	1.4	-26.5
	(1548 to 2275)	(-18.9 to 21.4)	(-37.1 to -6.9)	(19188 to 26857)	(-20 to 14.7)	(-39.6 to -13.5)	(716 to 901)	(-14.1 to 20.9)	(-37.8 to -12.5)
United Arab	182	123.2	-13.6	2132	118.8	-17.2	28	111	-17.8
Emirates	(126 to 259)	(66.3 to 192.9)	(-31.1 to 7.1)	(1526 to 2903)	(70.1 to 176.5)	(-33.8 to 1.9)	(23 to 35)	(66.4 to 163.1)	(-34 to 1.1)
Yemen	684	36.6	-10.2	9648	28.8	-14.3	215	29.5	-13.1
	(401 to 992)	(8.9 to 77)	(-27.3 to 13.9)	(5877 to 13924)	(4.8 to 62)	(-29.3 to 6.7)	(134 to 301)	(7 to 60.4)	(-27.8 to 6.9)
**South Asia**	**117544**	**36.9**	**4.0**	**1769489**	**32.3**	**-0.7**	**38774**	**36.5**	**-0.5**
	**(107899 to 136180)**	**(25.4 to 48.4)**	**(-4.3 to 12.5)**	**(1620354 to 2099212)**	**(22.0 to 42.9)**	**(-8.4 to 7.3)**	**(35483 to 45926)**	**(26.5 to 47.1)**	**(-7.9 to 7.2)**
Bangladesh	8873	8.6	-21.7	127944	4.4	-27	3304	11.8	-25.3
	(6278 to 12269)	(-15.0 to 36.7)	(-37.8 to -2.2)	(89564 to 170935)	(-16.3 to 29.2)	(-40.9 to -9.3)	(2293 to 3907)	(-9.3 to 38.0)	(-39.1 to -7.4)
Bhutan	44	30.0	-16.9	605	15.1	-23.6	15	14.6	-22.6
	(26 to 64)	(-2.3 to 68.1)	(-35.9 to 5.9)	(385 to 852)	(-10.4 to 45.3)	(-40.1 to -4.2)	(11 to 20)	(-8.7 to 43.3)	(-37.7 to -4.0)
India	98979	41.4	8.9	1504989	36.7	4.1	32307	40.6	3.7
	(89368 to 120933)	(28.1 to 54.3)	(-1.4 to 18.5)	(1363845 to 1868222)	(24.2 to 48.3)	(-5.4 to 13)	(29462 to 40044)	(28.3 to 51.9)	(-5.4 to 12.0)
Nepal	1981	32.5	-1.3	30738	25.5	-6.9	707	29.4	-6.0

	(1342 to 2749)	(5.6 to 67)	(-21 to 23.4)	(21362 to 42197)	(1.5 to 57.2)	(-25 to 16.7)	(507 to 941)	(5.7 to 60.1)	(-22.9 to 15.2)
Pakistan	7667	24.3	-12.8	105213	17.7	-17.3	2440	18.3	-15.8
	(5389 to 10891)	(-9.3 to 64.6)	(-35 to 13.5)	(74541 to 147656)	(-12.5 to 52.2)	(-37.7 to 5.1)	(1967 to 3044)	(-10.4 to 50)	(-35.1 to 5.4)
**East Asia**	**113036**	**42.0**	**17.6**	**1423547**	**35.1**	**7.2**	**33281**	**44.5**	**8.9**
	**(73551 to 122613)**	**(7.9 to 54.5)**	**(-10.9 to 27.8)**	**(923640 to 1544767)**	**(1.3 to 46.1)**	**(-19.7 to 15.8)**	**(29153 to 34581)**	**(9.2 to 56.0)**	**(-17.5 to 17.6)**
China	106128	44.7	19.7	1341894	37.1	8.6	30826	47.0	10.6
	(66519 to 115354)	(8.6 to 57.8)	(-10.3 to 30.5)	(841256 to 1461438)	(1.7 to 48.8)	(-19.6 to 17.5)	(26723 to 32113)	(9.7 to 58.9)	(-17.3 to 19.6)
North Korea	3136	10.1	-2.3	38999	8.9	-5.6	1075	13.1	-5.2
	(2108 to 4369)	(-14.3 to 43.0)	(-24.4 to 26.0)	(26498 to 53576)	(-13 to 37.7)	(-24.5 to 18.8)	(733 to 1504)	(-7.8 to 39.8)	(-23.2 to 16.6)
Taiwan (province of China)	1951	-7.0	-25.7	19722	-10.9	-30.1	834	-4.3	-31.6
	(1713 to 2215)	(-18.3 to 5.7)	(-34.9 to -15.1)	(17825 to 21841)	(-20.2 to -1)	(-37.4 to -21.9)	(806 to 863)	(-13.9 to 5.6)	(-38.4 to -24.2)
**Oceania**	**2325**	**28.2**	**-5.4**	**32058**	**25.9**	**-7.8**	**639**	**27.6**	**-7.0**
	**(1583 to 3172)**	**(2.9 to 60.4)**	**(-21.3 to 14.7)**	**(22840 to 42233)**	**(5 to 53.3)**	**(-21.6 to 9.5)**	**(493 to 818)**	**(8.6 to 51.1)**	**(-19.0 to 7.2)**
American Samoa	5	14.1	10.5	53	15.3	7.4	1	19.3	3.8
	(4 to 6)	(-8.7 to 41)	(-10.5 to 34)	(43 to 63)	(-3.6 to 38.5)	(-9.5 to 28.1)	(1 to 1)	(1.4 to 41.5)	(-10.9 to 20.6)
Federated States of Micronesia	14	6.3	-3.9	191	3.8	-7.4	5	6.3	-7.4
	(8 to 21)	(-30.5 to 42.1)	(-36.2 to 26.6)	(115 to 281)	(-27.9 to 36.1)	(-34.2 to 20.2)	(4 to 7)	(-19.1 to 34.5)	(-27.2 to 15.5)
Fiji	196	10.5	-2.9	2594	7.9	-6.1	69	14.4	-4.0
	(101 to 256)	(-16.8 to 41.9)	(-25.8 to 22.6)	(1331 to 3332)	(-15.5 to 36.3)	(-26.4 to 18.6)	(38 to 76)	(-9.6 to 42.6)	(-23.1 to 18.4)
Guam	14	13.9	7.9	156	19.5	10	4	30.0	11.7
	(11 to 17)	(-9.6 to 39.7)	(-14.3 to 31.8)	(129 to 184)	(-0.7 to 42.7)	(-8.2 to 30.8)	(3 to 4)	(9.4 to 52.5)	(-5.9 to 30.7)
Kiribati	43	20.9	-2.2	665	18.6	-4.2	16	20.2	-3.1
	(31 to 57)	(-4.2 to 48.3)	(-21.1 to 18.4)	(489 to 878)	(-4.3 to 43.8)	(-21.3 to 15.1)	(13 to 20)	(-0.7 to 43.3)	(-18.8 to 14.9)
Marshall Islands	11	8.5	-11.7	138	5.2	-15.2	3	8.7	-14.3
	(6 to 15)	(-16.3 to 43.4)	(-30.3 to 12.2)	(75 to 192)	(-16.2 to 32.8)	(-31.1 to 5.1)	(2 to 4)	(-11.8 to 35.1)	(-28.2 to 3.7)

Northern Mariana Islands	7<br>(5 to 9)	-14.6<br>(-36 to 11.2)	-3.1<br>(-22.6 to 21.1)	77<br>(62 to 94)	-0.9<br>(-19.9 to 22.6)	-0.8<br>(-17.1 to 21.1)	2<br>(2 to 2)	17.6<br>(-3.3 to 43.6)	-2.0<br>(-16.6 to 16.5)
Papua New Guinea	1744<br>(1067 to 2549)	33.1<br>(0.8 to 78.0)	-7.9<br>(-27.5 to 18.2)	24107<br>(15563 to 33951)	30.5<br>(3.4 to 67.5)	-10.5<br>(-27.4 to 11.6)	448<br>(315 to 619)	32.0<br>(6.5 to 66.1)	-9.6<br>(-24.4 to 9.3)
Samoa	18<br>(12 to 25)	9.9<br>(-20.5 to 43.0)	1.2<br>(-26.5 to 30.4)	228<br>(152 to 310)	9.9<br>(-17.5 to 39.2)	-0.2<br>(-24.5 to 25.1)	6<br>(4 to 8)	13.2<br>(-12.1 to 38.5)	0.5<br>(-21.5 to 23.6)
Solomon Islands	90<br>(60 to 132)	30.8<br>(0.1 to 70.0)	-6.4<br>(-26.1 to 18.5)	1289<br>(899 to 1780)	26.8<br>(0.7 to 59.7)	-10<br>(-26.6 to 10.6)	26<br>(20 to 35)	26.4<br>(3.6 to 54.6)	-10.0<br>(-24.4 to 7.0)
Tonga	16<br>(12 to 20)	4.9<br>(-18.1 to 29.2)	-5.8<br>(-25.8 to 16.2)	207<br>(159 to 260)	2.3<br>(-18 to 23.9)	-9.1<br>(-26.9 to 9.9)	7<br>(6 to 8)	5.4<br>(-14.0 to 26.2)	-9.6<br>(-26.1 to 7.8)
Vanuatu	41<br>(24 to 76)	22.0<br>(-8.3 to 61.0)	-6.3<br>(-26.8 to 19.6)	588<br>(348 to 1080)	18.6<br>(-6.2 to 51.6)	-9.0<br>(-26.3 to 13.8)	13<br>(8 to 24)	23.3<br>(0.4 to 52.7)	-8.1<br>(-22.7 to 11.3)
**Southeast Asia**	**62047**<br>**(52363 to 69261)**	**9.0**<br>**(0.6 to 20.4)**	**-14.5**<br>**(-20.9 to -6.2)**	**806446**<br>**(674759 to 906591)**	**2.9**<br>**(-4.4 to 12.9)**	**-20.3**<br>**(-25.9 to -13.1)**	**23822**<br>**(19745 to 26867)**	**7.8**<br>**(0.5 to 17.1)**	**-19.2**<br>**(-24.6 to -12.7)**
Cambodia	1672<br>(1218 to 2229)	19.3<br>(-5.5 to 53.8)	-11.1<br>(-28.9 to 13.1)	24254<br>(17975 to 31645)	10.9<br>(-10.3 to 41.2)	-17.2<br>(-33 to 4.4)	648<br>(469 to 811)	16.9<br>(-5.1 to 47.2)	-15.6<br>(-30.6 to 4.8)
Indonesia	22497<br>(16102 to 26420)	6.0<br>(-3.4 to 18.5)	-15.9<br>(-23 to -7.2)	309308<br>(222647 to 361835)	0.8<br>(-7.6 to 12.6)	-21.1<br>(-27.5 to -12.7)	9086<br>(6568 to 10741)	5.7<br>(-2.7 to 16.5)	-18.1<br>(-24.6 to -10.2)
Laos	614<br>(386 to 824)	11.9<br>(-12.7 to 47.5)	-17.2<br>(-34.6 to 7.2)	8791<br>(5701 to 11642)	2.6<br>(-18.2 to 32.4)	-23.7<br>(-38.7 to -1.7)	246<br>(149 to 314)	3.7<br>(-16.0 to 32.6)	-23.1<br>(-37.3 to -3.9)
Malaysia	2457<br>(1848 to 3057)	44.8<br>(9.5 to 78.5)	10.2<br>(-15.6 to 35.4)	28525<br>(21659 to 34323)	36.3<br>(5.7 to 65.2)	3.0<br>(-19.3 to 24.3)	709<br>(643 to 776)	43.4<br>(13.8 to 71.9)	2.6<br>(-18.2 to 21.3)
Maldives	16<br>(13 to 20)	18.2<br>(-2.5 to 46.4)	-26.2<br>(-38.9 to -7.6)	174<br>(145 to 210)	7.8<br>(-11.1 to 33.1)	-32.1<br>(-44.4 to -14.7)	5<br>(4 to 6)	9.8<br>(-9.6 to 38.8)	-32.3<br>(-44.2 to -12.2)
Mauritius	106<br>(91 to 125)	1.1<br>(-14.1 to 20)	-16.3<br>(-28.9 to -1.3)	1259<br>(1107 to 1428)	-0.5<br>(-13.4 to 13.5)	-19.8<br>(-30.1 to -8.7)	43<br>(40 to 47)	7.3<br>(-6.2 to 21.9)	-18.3<br>(-28.6 to -7.2)

Myanmar	7658	-6.1	-25.0	109027	-13.7	-31.8	43	-8.8	-29.8
	(5427 to 10298)	(-27.7 to 25.3)	(-41.5 to -0.4)	(78630 to 145480)	(-32.1 to 14.6)	(-45.8 to -10)	(40 to 47)	(-27.5 to 18.3)	(-43.5 to -10.5)
Philippines	8023	36.8	9.1	100693	31.8	4.0	2108	36.0	3.4
	(6245 to 10126)	(4.7 to 75.8)	(-16.6 to 40.1)	(79545 to 125380)	(1.8 to 67.3)	(-19.4 to 31.5)	(1912 to 2303)	(5.9 to 71.1)	(-18.7 to 28.6)
Sri Lanka	985	12.0	-10.2	11418	2.8	-18.7	374	9.6	-18.2
	(706 to 1297)	(-16.9 to 49.5)	(-33.0 to 20.4)	(8045 to 14713)	(-25 to 32.1)	(-40.5 to 4.3)	(298 to 405)	(-19.1 to 38.1)	(-39 to 2.4)
Seychelles	24	20.2	-1.5	279	17.4	-7.1	8	17.3	-7.2
	(20 to 29)	(0.9 to 42.7)	(-17.0 to 17.3)	(233 to 322)	(-0.2 to 37.4)	(-20.7 to 8.3)	(7 to 9)	(1.4 to 37.1)	(-19.8 to 7.4)
Thailand	8448	-7.7	-25.6	94250	-11.2	-31.1	3372	-5.2	-32.3
	(6904 to 10577)	(-26.3 to 14.2)	(-40.7 to -7.9)	(78255 to 118103)	(-27.4 to 7.5)	(-43.4 to -16.9)	(2987 to 3830)	(-21.3 to 13.0)	(-43.5 to -19.4)
Timor-Leste	89	27.4	-3.3	1254	17.9	-10.4	33	23.6	-10.3
	(59 to 120)	(0.2 to 63)	(-23.5 to 22.4)	(856 to 1665)	(-5.8 to 49.2)	(-28 to 13.1)	(24 to 42)	(0.4 to 53.7)	(-25.7 to 10)
Vietnam	9375	21.6	-9.4	116150	14.2	-15.7	3430	17.6	-13.8
	(6992 to 12049)	(-3.3 to 50.9)	(-27.6 to 12.6)	(86659 to 147654)	(-9.2 to 42.1)	(-32.7 to 4.6)	(2663 to 4099)	(-5.5 to 45.9)	(-31.1 to 6.8)
**Central sub-**	**16071**	**21.3**	**-12.5**	**265271**	**17.0**	**-15.5**	**6419**	**18.3**	**-14.5**
**Saharan Africa**	**(11496 to 20128)**	**(3.8 to 43.2)**	**(-24.6 to 2.8)**	**(193429 to 332876)**	**(-0.5 to 38.5)**	**(-28.1 to -0.2)**	**(4620 to 7788)**	**(0.7 to 39.4)**	**(-26.6 to -0.1)**
Angola	3425	17.8	-22.1	53382	10.4	-26.8	1286	14.2	-25.4
	(2352 to 4498)	(-9.4 to 54.5)	(-39.0 to -0.2)	(37023 to 69657)	(-14.3 to 41.7)	(-41.9 to -6.9)	(908 to 1601)	(-9.6 to 45.9)	(-40.8 to -5.7)
Central African	835	0.1	-17.8	15873	3.5	-16.3	416	5.1	-15.4
Republic	(470 to 1199)	(-23.7 to 28.8)	(-36.4 to 4.2)	(9208 to 22547)	(-20.7 to 33.8)	(-35.2 to 6.7)	(250 to 594)	(-18.5 to 33.8)	(-32.9 to 5.4)
Congo	995	24.5	-13.6	15657	18.4	-18.2	364	18.0	-16.8
(Brazzaville)	(543 to 1488)	(-5.0 to 66.3)	(-32.3 to 11.4)	(8840 to 22798)	(-7.1 to 52.9)	(-35.1 to 3.4)	(231 to 480)	(-6.1 to 47.3)	(-33.4 to 2.7)
Democratic	10480	24.7	-8.0	175524	20.9	-10.6	4212	21.4	-10.0
Republic of the	(7127 to 13983)	(-0.2 to 58)	(-25.9 to 14.5)	(119578 to 235897)	(-3.3 to 49.8)	(-27.9 to 10.6)	(2922 to 5471)	(-1.8 to 50.2)	(-27.2 to 10.9)
Congo									
Equatorial	127	26.4	-17.4	1718	13.7	-24.2	42	14.9	-23.2

Guinea	(69 to 201)	(-10.9 to 81.8)	(-40.2 to 16.5)	(968 to 2711)	(-18.1 to 62.1)	(-45 to 5.9)	(24 to 65)	(-16.5 to 59.6)	(-43.1 to 6.9)
Gabon	208	4.3	-23.7	3117	-3.2	-29.1	98	-3.5	-27.6
	(134 to 287)	(-21.0 to 38.2)	(-41.3 to -0.1)	(2001 to 4185)	(-25.4 to 25.9)	(-45 to -7.9)	(63 to 130)	(-23.8 to 23.4)	(-42.4 to -7.8)
**Eastern sub-**	**39062**	**23.6**	**-13.5**	**646308**	**18.0**	**-16.9**	**15719**	**18.7**	**-16.5**
**Saharan Africa**	**(33250 to 46432)**	**(13.3 to 36.8)**	**(-20.3 to -5.0)**	**(549919 to 769675)**	**(8.9 to 29.7)**	**(-23.1 to -9.1)**	**(13108 to 18222)**	**(10.1 to 29.6)**	**(-22.4 to -8.7)**
Burundi	1177	20.9	-13.2	20598	17.3	-14.5	494	18.1	-13.4
	(843 to 1591)	(-4.5 to 52.4)	(-30.5 to 8.7)	(14886 to 27391)	(-6.4 to 45.8)	(-32.3 to 6.6)	(342 to 641)	(-6.0 to 47.3)	(-30.1 to 6.1)
Comoros	127	15.4	-12.0	2115	13.4	-14.4	54	16.8	-13.3
	(91 to 173)	(-6.6 to 44.9)	(-28.3 to 10.1)	(1527 to 2930)	(-7.6 to 39.7)	(-30.3 to 5)	(41 to 70)	(-4.9 to 42.5)	(-29.3 to 6.1)
Djibouti	160	41.0	-15.1	2598	32.0	-19.5	54	33.1	-18.4
	(99 to 254)	(7.3 to 88.2)	(-34.5 to 11.5)	(1611 to 4153)	(0.7 to 75.3)	(-37.8 to 6.5)	(34 to 82)	(2.8 to 76.5)	(-35.8 to 6.4)
Eritrea	1033	18.4	-12.8	18179	14.7	-15.1	444	16.7	-13.9
	(713 to 1480)	(-7.1 to 50)	(-30.7 to 7.6)	(12684 to 25657)	(-9.3 to 41.8)	(-31.7 to 3.3)	(315 to 602)	(-6 to 41.8)	(-29.6 to 4.3)
Ethiopia	7416	7.7	-24.8	122492	-1.4	-29.7	3688	-0.4	-29.4
	(5523 to 11738)	(-10.4 to 41.9)	(-36.5 to -2.1)	(92549 to 192761)	(-16.7 to 28.8)	(-40.3 to -9.3)	(2863 to 5556)	(-15.3 to 28.1)	(-39.4 to -10.1)
Kenya	3429	28.7	-12.2	53353	24.8	-15.1	1221	26.9	-13.5
	(2813 to 4828)	(14.8 to 50.1)	(-21.1 to 2.1)	(43698 to 76026)	(12.4 to 43.5)	(-23.3 to -2.6)	(922 to 1606)	(14.8 to 45.3)	(-21.6 to -1.3)
Madagascar	3415	33.6	-5.7	58152	32.4	-7.1	1206	33.1	-6.7
	(2441 to 4624)	(6.6 to 70.6)	(-24.6 to 20.0)	(41023 to 77528)	(5.3 to 68.4)	(-26.3 to 17.9)	(896 to 1515)	(5.9 to 68.5)	(-25.8 to 16.1)
Malawi	2042	25.4	-13.1	32291	19.4	-16.8	774	21.0	-15.9
	(1423 to 2799)	(-7.5 to 72.5)	(-34.4 to 18.2)	(23308 to 43429)	(-9.8 to 60.2)	(-36.6 to 10.5)	(590 to 976)	(-6.6 to 58.8)	(-34.9 to 10.2)
Mozambique	3955	29.4	-6.3	68061	24.6	-9.0	1543	24.0	-8.5
	(2503 to 5544)	(-0.5 to 71.0)	(-27.8 to 23.7)	(43193 to 93235)	(-4.7 to 61.9)	(-30.4 to 18.3)	(999 to 2073)	(-5.0 to 59.7)	(-30.3 to 17.7)
Rwanda	1426	21.9	-17.4	23079	13.9	-22.3	581	18.2	-21.1
	(1035 to 2025)	(-4.7 to 55.4)	(-35.1 to 3.7)	(16648 to 31790)	(-8.9 to 40.8)	(-37.7 to -4)	(448 to 751)	(-4.7 to 46.1)	(-35.8 to -2.9)
Somalia	2527	28.2	-6.4	45546	21.2	-9.2	1060	22.5	-8.3

	(1626 to 3736)	(2.8 to 65.5)	(-24.5 to 19.3)	(29214 to 66654)	(-2.4 to 55.9)	(-26.6 to 15.6)	(712 to 1444)	(-0.8 to 56)	(-25.3 to 14.9)
South Sudan	1292	21.4	-5.8	21822	22.3	-7.1	482	23.5	-7.2
	(860 to 1851)	(-4.7 to 59.8)	(-25.5 to 22.4)	(14864 to 30907)	(-2.5 to 59.7)	(-26.4 to 20.8)	(313 to 682)	(-1.3 to 60.1)	(-25.6 to 19.2)
Tanzania	5930	32.6	-5.5	94738	28.0	-9.0	2188	27.5	-8.5
	(4281 to 8045)	(0.8 to 74.6)	(-26.5 to 21.9)	(69970 to 126173)	(-0.4 to 63.8)	(-28.5 to 16.5)	(1617 to 3104)	(0.6 to 61.3)	(-27.5 to 14.2)
Uganda	3124	12.8	-22.1	50060	9.7	-24.3	1291	11.2	-23.9
	(2300 to 4080)	(-13.3 to 45.6)	(-38.7 to -2.2)	(38353 to 64203)	(-13.7 to 37.4)	(-40.4 to -5.0)	(1035 to 1558)	(-11.9 to 38.8)	(-38.9 to -4.9)
Zambia	1986	55.0	-0.1	32818	46.1	-5.2	629	47.2	-4.5
	(1433 to 2578)	(17.5 to 103.6)	(-24.7 to 30.6)	(23950 to 41708)	(11.1 to 92.0)	(-28.1 to 24.0)	(478 to 828)	(12.8 to 91.2)	(-27.3 to 22.7)
**Southern sub-**	**12498**	**-17.8**	**-32.3**	**182929**	**-17.6**	**-32.7**	**6582**	**-10.7**	**-28.5**
**Saharan Africa**	**(11079 to 13588)**	**(-29.9 to -7.0)**	**(-41.6 to -24.0)**	**(162744 to 199885)**	**(-27.5 to -7.8)**	**(-40.4 to -25.1)**	**(6057 to 7431)**	**(-19.8 to -1.0)**	**(-35.2 to -21)**
Botswana	312	43.3	0.9	4391	32.9	-6.1	108	29.7	-4.7
	(240 to 386)	(-17.8 to 120.4)	(-41.1 to 51.7)	(3428 to 5356)	(-22.2 to 101.1)	(-44.8 to 41.8)	(75 to 169)	(-22.7 to 91.2)	(-43 to 39.8)
Lesotho	373	-6.8	-15.3	6286	-9.3	-17.2	229	-8.8	-15.5
	(238 to 541)	(-35.9 to 31.1)	(-41.1 to 18.4)	(3987 to 8910)	(-37.6 to 28.9)	(-43 to 17.0)	(163 to 307)	(-35.7 to 26.5)	(-39.9 to 16.1)
Namibia	229	3.3	-22.8	3444	-3.1	-27.9	115	-1.9	-27.6
	(161 to 318)	(-25.2 to 48.3)	(-43.9 to 9.8)	(2513 to 4715)	(-29.9 to 37.7)	(-47.9 to 1.4)	(83 to 149)	(-28.5 to 35)	(-47.1 to -0.4)
South Africa	9327	-23.1	-36.0	129706	-22.6	-36.1	4914	-14.1	-30.8
	(8264 to 10145)	(-36 to -11.1)	(-46.3 to -26.4)	(116256 to 140314)	(-34.1 to -11.7)	(-45.1 to -27.3)	(4587 to 5601)	(-24.4 to -2.4)	(-38.8 to -21.6)
Swaziland	163	-13.8	-28.0	2536	-19.5	-32.1	94	-15.3	-30.1
(eSwatini)	(106 to 239)	(-38.5 to 22.5)	(-47.5 to -0.3)	(1631 to 3690)	(-41.8 to 12.6)	(-50.3 to -6.6)	(58 to 129)	(-37.2 to 15.7)	(-47.6 to -6.2)
Zimbabwe	2095	1.7	-23.2	36565	-2.1	-26.0	1122	-0.8	-24.6
	(1540 to 2727)	(-21.5 to 30.6)	(-39.8 to -3.0)	(26892 to 47334)	(-23.1 to 23.5)	(-41.7 to -7.8)	(891 to 1354)	(-22.1 to 23.1)	(-40.3 to -7)
**Western sub-**	**39955**	**30.2**	**-8.0**	**611846**	**28.6**	**-10.2**	**13637**	**29.8**	**-9.2**
**Saharan Africa**	**(31011 to 49933)**	**(12.6 to 51.5)**	**(-18.9 to 6.2)**	**(480371 to 753733)**	**(13.7 to 47.7)**	**(-19.9 to 2.8)**	**(10774 to 16838)**	**(16.2 to 48.4)**	**(-18.4 to 3.1)**
Benin	1120	36.3	-4.1	18117	36.5	-5.0	393	36.5	-4.2

	(767 to 1481)	(5.1 to 72.6)	(-24.7 to 18.9)	(12450 to 24044)	(7.2 to 73.0)	(-25.1 to 19.6)	(290 to 504)	(7.9 to 70.4)	(-24 to 19.7)
Burkina Faso	2601	40.9	-1.2	41434	38.9	-2.4	857	40.0	-0.9
	(1852 to 3406)	(12 to 76.8)	(-20.1 to 22.5)	(29346 to 53314)	(13.4 to 71.0)	(-19.9 to 19.2)	(649 to 1048)	(15.9 to 70.7)	(-17.2 to 20.7)
Cameroon	2671	20.6	-18.2	42277	16.9	-20.3	1044	17.7	-18.7
	(1626 to 3767)	(-7.3 to 57.2)	(-36.2 to 5.7)	(25627 to 59406)	(-9.7 to 49.1)	(-38.3 to 2.0)	(712 to 1356)	(-8.6 to 49.4)	(-36.2 to 3)
Cape Verde	54	37.7	7.4	715	27.6	-0.3	20	27.8	0.0
	(45 to 73)	(11.7 to 67.1)	(-12.5 to 29.1)	(603 to 931)	(3.8 to 50.2)	(-18.7 to 16.8)	(18 to 25)	(6.0 to 49.6)	(-16.8 to 16.5)
Chad	1443	32.7	-2.0	24638	30.7	-3.5	554	28.9	-2.3
	(996 to 1930)	(3.7 to 67.9)	(-22.4 to 22.6)	(17020 to 32902)	(3.7 to 63.1)	(-23.6 to 19.9)	(415 to 702)	(2.9 to 60.2)	(-22.6 to 21.2)
Côte d’Ivoire	1089	20.0	-10.9	18719	16.2	-13.5	456	17.2	-13.2
	(793 to 1459)	(-6.5 to 63.4)	(-30.3 to 20.1)	(13869 to 13869)	(-9.2 to 57.3)	(-32.4 to 15.1)	(342 to 581)	(-7.8 to 55.9)	(-31.2 to 13.6)
The Gambia	169	31.6	-4.6	2655	28.4	-6.6	60	27.6	-6.9
	(126 to 231)	(0.6 to 67.2)	(-26 to 20.6)	(1991 to 3540)	(1.0 to 60.3)	(-27.4 to 17.6)	(47 to 75)	(-0.2 to 58.8)	(-27 to 17.0)
Ghana	3693	34.0	-6.9	54367	26.8	-11.4	1224	28.3	-10.3
	(2408 to 4956)	(4.4 to 73.3)	(-26.8 to 18.9)	(35457 to 72475)	(-0.5 to 60.7)	(-30.3 to 12.4)	(812 to 1574)	(1.8 to 62.1)	(-28.4 to 12.6)
Guinea	1955	18.1	-5.7	32535	15.3	-6.7	848	9.9	-6.0
	(1486 to 2506)	(-6.6 to 46.4)	(-25.4 to 16.9)	(24646 to 41305)	(-6.7 to 41.6)	(-24.3 to 14.8)	(680 to 1025)	(-10.2 to 34)	(-22.9 to 14.0)
Guinea-Bissau	251	24.0.	-7.8	4178	18.9	-10.4	98	19.1	-8.6
	(158 to 352)	(-4.4 to 58.7)	(-27.2 to 15)	(2685 to 5729)	(-6.0 to 49.7)	(-28.4 to 10.9)	(68 to 126)	(-4.5 to 47.5)	(-26.7 to 12.8)
Liberia	462	38.0	-3.5	7406	36.6	-4.5	159	33.7	-3.0
	(306 to 626)	(9.2 to 74.8)	(-22.7 to 19.2)	(4920 to 9956)	(9.2 to 69.4)	(-22.5 to 18.5)	(111 to 204)	(8.1 to 63.6)	(-20.6 to 18.1)
Mali	1437	27.8	-8.6	22262	22.0	-11.8	509	23.6	-10.7
	(1010 to 2093)	(-0.2 to 61.6)	(-27.7 to 13.1)	(16137 to 32257)	(-2.3 to 50.7)	(-29.4 to 7.9)	(395 to 673)	(-0.2 to 50.4)	(-27.2 to 8.2)
Mauritania	414	24.0	-7.0	6220	18.7	-11.4	161	20.6	-10.3
	(295 to 546)	(-1.5 to 53.6)	(-25.6 to 14.5)	(4387 to 8145)	(-4.7 to 45.0)	(-28.9 to 8.8)	(120 to 198)	(-3.0 to 47.0)	(-27.3 to 8.9)
Niger	1951	34.8	-3.6	32787	34.9	-4.7	686	40.1	-3.1

	(1310 to 2626)	(6.4 to 70.7)	(-22.3 to 20.2)	(22548 to 43502)	(8 to 68.5)	(-23.2 to 18.0)	(507 to 902)	(12.8 to 74)	(-21 to 19.6)
Nigeria	17265	30.8	-8.7	248421	32	-10.5	5314	34.1	-9.5
	(10762 to 26399)	(-3.4 to 84.7)	(-31.0 to 26.3)	(154709 to 377202)	(0.9 to 83.8)	(-30.7 to 23.1)	(3110 to 7945)	(4.3 to 84.2)	(-28.7 to 22.0)
São Tomé and	30	23.3	-7.9	442	22.0	-9.9	10	23.7	-6.7
Príncipe	(19 to 42)	(-2.7 to 56.1)	(-26.3 to 14.4)	(280 to 600)	(0.2 to 50.6)	(-25.6 to 11.2)	(7 to 14)	(2.2 to 51.2)	(-22.8 to 13.3)
Senegal	1596	30.6	-0.1	26623	28.6	-2.2	619	31.3	-1.0
	(1185 to 2061)	(3.2 to 61.6)	(-19.8 to 22.3)	(20057 to 33762)	(2.8 to 56.6)	(-20.8 to 18.9)	(494 to 741)	(7.7 to 58.5)	(-18.3 to 19.4)
Sierra Leone	908	24.8	-6.3	14529	24.4	-7.2	337	25.4	-5.6
	(577 to 1234)	(0.4 to 56.0)	(-24.4 to 16.9)	(9187 to 19621)	(-0.7 to 55.4)	(-25.4 to 15.8)	(236 to 427)	(1.3 to 55.2)	(-23.3 to 16.7)
Togo	845	38.6	-4.1	13517	34	-7.6	286	37.3	-7.1
	(564 to 1146)	(3.9 to 82.0)	(-27.3 to 25.6)	(9289 to 18299)	(2.3 to 75.3)	(-29.1 to 21.8)	(206 to 372)	(5.1 to 80.7)	(-28.0 to 19.9)

UI= 95% uncertainty intervals. DALYs=disability-adjusted life-years. SDI=Socio-demographic Index.
