# Supplementary material for: Global, regional, and national burden of cervical cancer for 195 countries and territories, 2007–2017: findings from the Global Burden of Disease Study 2017
Source: BMC Womens Health. 2021 Dec 18;21:419. doi: 10.1186/s12905-021-01571-3 (PMC8684284; doi:10.1186/s12905-021-01571-3)
Supplement: Supplementary file 3 — Additional file 3: Fig. S1. Age-specific incidence (A), DALYs (B), and death (C) rates of cervical cancer by SDI quintiles. [file 12905_2021_1571_MOESM3_ESM.pdf]

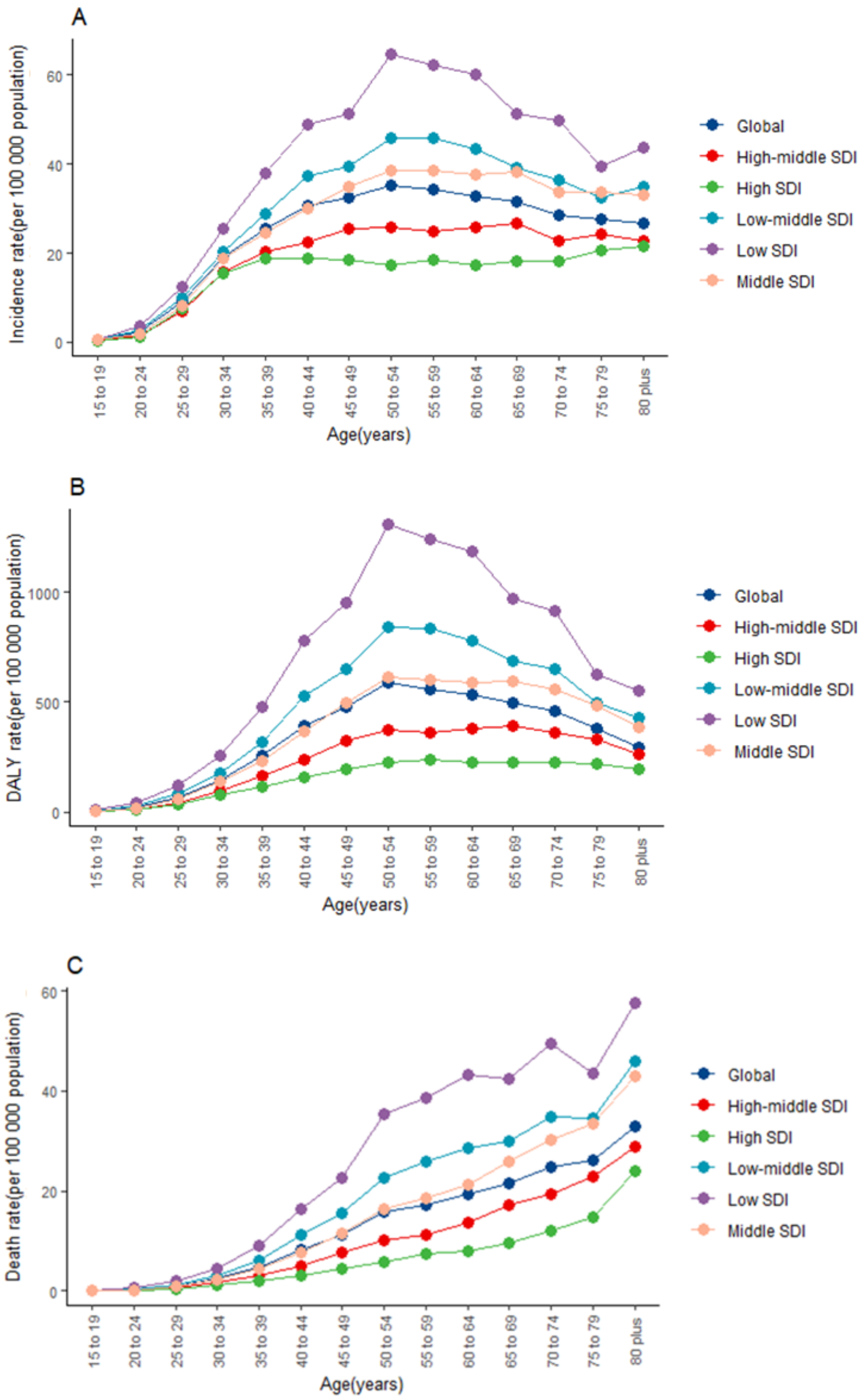

Figure S1: Age-specific incidence(A), DALYs(B), and death(C) rates of cervical cancer by SDI quintiles.

DALYs=disability-adjusted life-years. SDI= Socio-demographic Index.
